# Exosomal miR‐3126‐5p derived from cancer‐associated fibroblasts facilitates glycolysis to accelerate NSCLC progression by targeting KLF13 to activate the SH2B1/IRS1 axis

**DOI:** 10.1002/ctm2.70554

**Published:** 2025-12-18

**Authors:** Zhenyu Zhang, Haicheng Ma, Yingying Zheng, Lina Wang, Chenghui Wang, Yuanyuan Liu, Hengxiao Lu, Shaoqiang Wang

**Affiliations:** ^1^ Weifang People's Hospital, Shandong Second Medical University Weifang Shandong P.R. China; ^2^ Health Management Center Weifang People's Hospital, Shandong Second Medical University Weifang Shandong P.R. China; ^3^ Medical Research Center Affiliated Hospital of Jining Medical University, Jining Medical University Jining Shandong P. R. China; ^4^ Department of Pathology Affiliated Hospital of Jining Medical University, Jining Medical University Jining Shandong P. R. China; ^5^ Clinical laboratory Affiliated Hospital of Jining Medical University, Jining Medical University Jining Shandong P. R. China; ^6^ Department of Thoracic Surgery Weifang People's Hospital, Weifang Medical University Weifang Shandong P. R. China; ^7^ Shandong Provincial Key Medical and Health Laboratory of BT and IT for Thoracic Oncology (Weifang People's Hospital) Weifang Shandong P. R. China

**Keywords:** cancer‐associated fibroblasts, exosomal miR‐3126‐5p, glycolysis, KLF13, non‐small‐cell lung cancer, SH2B1

## Abstract

**Background:**

As a critical component of the tumour microenvironment, cancer‐associated fibroblasts (CAFs) actively drive the malignant advancement of non‐small‐cell lung cancer (NSCLC); however, their underlying mechanisms continue to be poorly characterized. This work examined the role of CAFs‐derived exosomal miR‐3126‐5p in the glycolysis of NSCLC cells.

**Methods:**

Glycolysis was evaluated by lactate production, glucose uptake, oxygen consumption rate (OCR) and extracellular acidification rate (ECAR). Cell proliferation and cycle were evaluated by CCK‐8, EdU staining, and flow cytometry. Src homology 2B adaptor protein 1 (SH2B1) and insulin receptor substrate 1 (IRS1) protein interaction was tested by Co‐IP and GST pull‐down assay. ChIP, dual‐luciferase reporter assay, and EMSA determined the binding of kruppel‐like factor 13 (KLF13) to the SH2B1 promoter. Dual‐luciferase reporter assay was applied to assess miR‐3126‐5p binding to KLF13 3′‐UTR. In vivo growth of NSCLC was determined in the mouse xenograft and Lewis lung carcinoma models.

**Results:**

CAFs‐derived exosomal miR‐3126‐5p was highly expressed in NSCLC tissues, and its elevated plasma level was significantly associated with poor prognosis of NSCLC patients. CAFs‐derived exosomal miR‐3126‐5p facilitated glycolysis to accelerate the malignant progression of NSCLC cells. KLF13 exhibited reduced expression in NSCLC, while its overexpression suppressed NSCLC growth via repressing glycolysis. Exosomal miR‐3126‐5p targeted KLF13 3′‐UTR to inhibit its expression in NSCLC cells. KLF13 transcriptionally inhibited SH2B1 expression to abolish the interaction between SH2B1 and IRS1 proteins, thus repressing PI3K/AKT pathway‐mediated glycolysis. KLF13 knockdown counteracted the anti‐cancer action of exosomal miR‐3126‐5p inhibition.

**Conclusion:**

CAFs‐derived exosomal miR‐3126‐5p accelerated NSCLC progression via inhibiting KLF13 expression, which transcriptionally activated SH2B1 to promote its interaction with IRS1, thereby promoting PI3K/AKT pathway‐mediated glycolysis. Our findings position CAFs‐secreted exosomal miR‐3126‐5p as a novel therapeutic intervention with potential in NSCLC management.

**Highlights:**

- CAFs‐derived exosomal miR‐3126‐5p enhanced glycolysis of NSCLC cells via targeting KLF13.
- KLF13 led to transcriptional inhibition of SH2B1 in NSCLC cells.
- SH2B1 interplayed with IRS1 to facilitate glycolysis of NSCLC cells.
- IRS1 promoted glycolysis of NSCLC cells via the activation of PI3K/AKT pathway.

## Graphical abstract

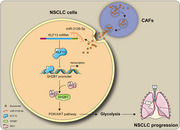

## 1 INTRODUCTION

Lung cancer leads as the primary driver of cancer‐related death globally, which has become a huge public health challenge.^1^ Non‐small‐cell lung cancer (NSCLC) accounts for the largest subgroup of this disease, possessing the highest mortality rate.^2^ Despite advances in clinical therapeutics, the prognosis of NSCLC remains pessimistic owing to postoperative recurrence.^3^ Unlike normal cells, cancer cells with a rapid rate of proliferation exhibit a great elevation in glucose uptake and lactate production during the process known as aerobic glycolysis.^4^ Recent studies have suggested glycolysis as a pivotal driver for NSCLC development.^5^, ^6^ A recent study documented that HVEM interplayed with MPRIP protein to repress glycolysis, thus delaying NSCLC progression.^7^ Another work reported that inactivation of lncRNA AC016727.1/BACH1/HIF‐1α loop suppressed NSCLC growth and metastasis via inhibiting glycolysis.^8^ However, molecular mechanisms of glycolysis regulation in NSCLC cells remain largely unelucidated.

The tumour microenvironment, which provides the dynamic interplays between cancer cells and their surrounding components, exerts a pivotal impact on the glycolysis of tumours.^9^, ^10^ Cancer‐associated fibroblasts (CAFs) constitute a vital component of the tumour microenvironment, which play key roles in the malignant development of NSCLC cells.^11^ It has been indicated that CAFs exert their carcinogenic functions by releasing exosomes (Exos) that contain proteins, RNAs, non‐coding RNAs, and so on.^12^ Previous studies revealed that CAFs‐derived Exos contributed to glycolysis of various human cancers, such as breast cancer^13^ and liver cancer.^14^ MicroRNAs, a subset of noncoding RNAs containing 22–26 nucleotides, can repress the transcription of mRNAs. MiRNAs‐loaded Exos released from CAFs were shown to serve as key regulators of NSCLC malignancy.^15^ Until now, it is still unclear how the miRNAs‐loaded Exos derived from CAFs precisely regulate the glycolysis in NSCLC.

Src homology 2B adaptor protein 1 (SH2B1) belongs to the SH2B family, which exerts key roles in glucose and lipid metabolism.^16^ A previous study reported that SH2B1 knockout led to metabolic disorders, such as hyperglycemia, hyperlipidemia, and obesity in mice,^17^ suggesting the crucial role of SH2B1 in the maintenance of glucose homeostasis. Previous studies documented that SH2B1 was overexpressed in NSCLC, which actively enhanced both growth and metastatic progression of NSCLC cells.^18^, ^19^ However, the modulation of SH2B1 in glycolysis in the context of NSCLC remains unknown. Besides, our previous study reported that SH2B1 could interact with insulin receptor substrate 1 (IRS1) to increase its expression, which facilitated epithelial–mesenchymal transition of lung adenocarcinoma cells.^20^ IRS1 is a key member of the insulin receptor substrate family that has been reported to modulate glucose metabolism via PI3K/AKT pathway in diabetes‐related hepatic steatosis^21^ and insulin‐resistant HepG2 cell model.^22^ Wang et al.^23^ documented that PYCR1 knockdown inhibited IRS1‐mediated glycolysis of HCC cells, suggesting the promotive role of IRS1 in glycolysis. So far, whether SH2B1 influences NSCLC cell glycolysis by orchestrating the IRS1/PI3K/AKT pathway lacks definitive experimental evidence.

It has been reported that transcription factors integrate stress signals to serve as key regulators of glycolysis in cancers.^24^, ^25^ Kruppel‐like factor 13 (KLF13) functions as a transcription factor that plays the inhibitory effect on transcription of its target genes.^26^ Mounting evidence has proved the biological function of KLF13 in various tumours. For instance, KLF13 was demonstrated to suppress the progression of thyroid carcinoma via inhibiting IFIT1 expression.^27^ Another study showed that KLF13 acted as a tumour suppressor of colorectal cancer via transcriptional inhibition of HMGCS1.^28^ The TCGA database uncovered reduced KLF13 levels in lung cancer, which was linked to diminished overall survival rate. However, the influence of KLF13 on NSCLC, as well as glycolysis, remains unclear. As predicted by the JASPAR database, KLF13 possessed binding sites to the promoter of SH2B1. Therefore, KLF13 might affect glycolysis during NSCLC progression via modulation of transcriptional activity of SH2B1, which deserves to be validated.

In addition, the ENCORI database predicted miR‐3126‐5p as one of the top 10 miRNAs with binding sites to the 3′‐UTR of KLF13 mRNA. Our preliminary experiment firstly discovered that miR‐3126‐5p was upregulated in CAFs and could negatively regulate KLF13 expression. Up to now, only one research study disclosed miR‐3126‐5p was upregulated in gefitinib‐resistant lung cancer cells,^29^ suggesting the potential contribution of miR‐3126‐5p to lung cancer progression. So far, the regulation of miR‐3126‐5p in glycolysis has not been reported. Furthermore, whether CAFs‐derived exosomal miR‐3126‐5p might promote glycolysis during the malignant progression of NSCLC cells by targeting KLF13 remains unresolved.

The present work identified that CAFs‐derived Exos transferred miR‐3126‐5p to NSCLC cells, which targeted and inhibited KLF13 expression to facilitate SH2B1 transcription, and subsequently interacted with IRS1 to activate the PI3K/AKT pathway, thereby promoting glycolysis to drive NSCLC progression. Our data point to CAFs‐derived exosomal miR‐3126‐5p as an emerging therapeutic candidate for NSCLC, which enhances the possibility of intervening in the metabolic reprogramming to delay NSCLC progression via the tumour microenvironment.

## 2 MATERIALS AND METHODS

### 2.1 Clinical samples

Fresh excised NSCLC tissues and paired normal para‐cancerous lung tissues were procured from 40 patients from the Affiliated Hospital of Jining Medical University (Jining, Shandong, China) during surgical procedures. The clinical tissues histologically identified as NSCLC were incorporated and immediately stored in liquid nitrogen. Peripheral blood samples were collected from 20 healthy donors and the 40 NSCLC patients mentioned above. All patients did not receive chemotherapy or radiotherapy before inclusion, and informed consent was collected from all participants.

### 2.2 Cell culture

NSCLC cells, including A549 and NCI‐H1299, were sourced from the American Type Culture Collection (ATCC, Manassas, USA) and maintained in RPMI‐1640 (Gibco, New York, USA) with 10% fetal bovine serum (FBS, Gibco) at 37°C with 5% CO_2_. SH2B1‐knockout A549 and NCI‐H1299 cells were acquired from Haixing Biosciences. Lewis lung carcinoma LLC cells were sourced from Procell and cultured with DMEM (Gibco) supplemented with 10% FBS.

### 2.3 Isolation of primary normal fibroblasts (NFs)/CAFs and identification

CAFs and NFs were extracted from NSCLC samples and normal lung tissues as previously described.^30^ Briefly, the tissues were physically minced, followed by digestion with DNase (2 mg/mL, Gibco) and collagenase type I (10 mg/mL, Gibco) for 1.5 h at 37°C. After filtration and centrifugation, the primary CAFs and NFs were isolated and maintained in DMEM supplemented with 15% FBS, 20 ng/mL EGF (MedChemExpress, Monmouth Junction, USA), and 1% penicillin/streptomycin, propagated at 37°C in a humidified incubator. The primary CAFs and NFs at passages 4–10 were used in the following experiments. CAFs were identified by positive expression of fibroblast markers (α‐SMA, vimentin, and FAP) by western blotting and immunofluorescence staining.

### 2.4 Cell transfection

Short hairpin RNAs designed to silence SH2B1 (sh‐SH2B1#1‐3) and IRS1 (sh‐IRS1), negative control (NC) of shRNA (sh‐NC), SH2B1 overexpression construct and its corresponding negative control plasmid were procured from GeneChem (Shanghai, China). A549 and NCI‐H1299 cells (5 × 10^5^ cells per well) underwent transfection with these vectors via Lipofectamine 3000 (Thermo Fisher Scientific, Waltham, MA, USA).

Lentiviruses carrying sh‐NC, sh‐KLF13, vector, KLF13, miR‐3126‐5p mimics, and miR‐3126‐5p inhibitor were packaged and purchased from GeneChem. For lentivirus infection, lung cancer cells, CAFs, or LLC cells were transduced with lentiviruses containing 5 µg/mL polybrene (MedChemExpress), followed by subsequent selection with 2 µg/mL puromycin (MedChemExpress).

### 2.5 5‐Ethynyl‐2′‐deoxyuridine staining

NSCLC cells were inoculated into 24‐well plates with 80% confluence. The cells were labelled with 20 µM 5‐ethynyl‐2′‐deoxyuridine (EdU) (Abcam, Cambridge, UK) for 2 h after recovery overnight. After fixation and permeabilization, the cells were reacted with EdU Additive Solution for 45 min. EdU‐positive cells were examined under a fluorescence microscope (Olympus, Tokyo, Japan).

### 2.6 Cell counting kit‐8

NSCLC cells (5 × 10^3^/well) were planted into 96‐well plates. After multiple treatments, 15 µL of cell counting kit‐8 (CCK‐8) solution (HY‐K0301, MedChemExpress) was dispensed into each well and then incubated for 2 h of incubation at 37°C. The microplate reader (Thermo Fisher Scientific) was utilized to assess absorbance at 450 nm.

### 2.7 Flow cytometry

Cell cycle distribution was evaluated by the DNA content quantitation assay (CA1510, Solarbio, Beijing, China). NSCLC cells were inoculated into six‐well plates (2 × 10^5^ cells/well). Following fixation with 500 µL of precooled 70% ethanol at 4°C overnight, the cells were exposed to 150 µL of RNase A solution for 45 min. Then, staining with 350 µL of propidium iodide (PI) solution was conducted for 30 min in a dark place at 4°C. Cell cycle was detected on a flow cytometer (Thermo Fisher Scientific).

### 2.8 Glucose uptake and lactate production measurement

The glucose uptake assay kit (ab136955, Abcam) was applied to determine glucose uptake. After processing, the samples were reacted with the working solution according to the protocol. The absorbance at 412 nm was determined with a microplate reader. The lactic acid content assay kit (BC2235, Solarbio) was used to analyze the lactate level in tissue and cell samples, following the manufacturer's protocol.

### 2.9 Detection of oxygen consumption rate and extracellular acidification rate

The XF96 extracellular flux analyzer (Agilent Seahorse, Santa Clara, USA) was utilized to assess oxygen consumption rate (OCR) and extracellular acidification rate (ECAR). NSCLC cells (12,000 cells/well) were planted into the XF96 cell culture plates and cultured for 2 h at 37°C for attachment. The OCR and ECAR were determined using the Cell Mito stress test kit (103015‐100, Agilent Seahorse) and the Glycolytic rate assay kit (103344‐100, Agilent Seahorse), respectively. Finally, the results obtained were processed using Seahorse XFp Wave software.

### 2.10 Bioinformatics analysis

JASPAR database (https://jaspar.elixir.no/) was applied to predict the binding of KLF13 to the SH2B1 promoter. ENCORI database (https://rnasysu.com/encori/) was adopted to predict the miRNAs possessing binding sites to the 3′‐UTR of KLF13 mRNA. The differential expression of KLF13 in LUSC and LUAD was evaluated by GEPIA (http://gepia.cancer‐pku.cn/). For LUAD, the number for tumour = 483; the number for normal = 347. For LUSC, the number for tumour = 486; the number for normal = 338. The association between KLF13 expression and 5‐year overall survival in lung cancer patients was analyzed utilizing the Kaplan–Meier plotter (https://kmplot.com/analysis/). Number for KLF13 low expression group = 706; number for KLF13 high expression group = 705.

### 2.11 Co‐immunoprecipitation

Co‐immunoprecipitation (Co‐IP) was exploited to evaluate the interaction between SH2B1 and IRS1 proteins utilizing the Magnetic IP/Co‐IP kit (88804, Thermo Fisher Scientific). NSCLC cells were lysed with immunoprecipitation buffer (IP buffer, Beyotime, Haimen, China). Cell lysates were immunoprecipitated by anti‐SH2B1 (12226‐1‐AP, Proteintech, Wuhan, China) or non‐specific IgG antibody (ab172730, Abcam) for 12 h at 4°C. Subsequently, cell lysates were mixed with Protein A/G beads and incubated at 4°C for 2 h. After washing with the IP buffer, the immunoprecipitated proteins were determined by western blotting.

### 2.12 GST pull‐down assay

The direct interaction between SH2B1 and IRS1 proteins was evaluated using a GST pull‐down assay. Purified His‐IRS1 was incubated with either GST‐SH2B1 or GST protein (negative control) in binding buffer at 4°C for 2 h. Subsequently, the mixtures were incubated with glutathione‐sepharose beads at 4°C for 1 h. The beads were then washed extensively with binding buffer, and the bound proteins were eluted by boiling in SDS‐PAGE loading buffer. Finally, the eluted proteins were separated by SDS‐PAGE and analyzed by western blotting using anti‐His and anti‐GST antibodies.

### 2.13 Electrophoretic mobility shift

Biotin‐labelled SH2B1 probe, specific competitive probe, and unspecific probe were engineered and produced by GenePharma (Shanghai, China). Briefly, the KLF13 protein samples were incubated with the biotin‐labelled SH2B1 probe, competitive probe, or unspecific probe for 20 min. After that, the complexes were subjected to non‐denaturing polyacrylamide gel electrophoresis. The biotinylated DNA was detected with the chemiluminescent electrophoretic mobility shift (EMSA) kit (20148, Thermo Fisher Scientific).

### 2.14 Chromatin immunoprecipitation

Chromatin immunoprecipitation (ChIP) assay was implemented with the Pierce Magnetic ChIP kit (26157, Thermo Fisher Scientific). NSCLC cells were cross‐linked by 1% paraformaldehyde. Then, the chromatins were subjected to sonication to produce 200–800 bp fragments. Immunoprecipitation was conducted using anti‐KLF13 (PA5‐80754, Thermo Fisher Scientific) or anti‐IgG (ab172730, Abcam). Subsequently, the mixture was treated with Protein A/G magnetic beads for 4 h. After treatment with RNase A and proteinase K, the immunoprecipitated DNA was detected by qPCR.

### 2.15 Dual‐luciferase reporter assay

To validate the interaction of miR‐3126‐5p with the 3′‐UTR of KLF13 mRNA, the wild type (WT) and mutant (MUT) sequences for KLF13 3′‐UTR were cloned into the pmirGLO vector (Promega, Madison, USA). To evaluate the binding of KLF13 to the SH2B1 promoter, the WT or MUT sequences for the SH2B1 promoter were inserted into the pGL3‐basic vector (Promega). NSCLC cells were transfected with the luciferase constructs together with sh‐KLF13, sh‐NC, miR‐3126‐5p mimics, mimics NC, or pRL‐TK (Promega) by Lipofectamine 3000. The relative luciferase activity was detected using the dual‐luciferase reporter assay system (E1910, Promega) following 48 h of incubation.

### 2.16 Isolation and identification of Exos

Exos were extracted from CAFs/NFs culture supernatants and peripheral blood plasma utilizing the total exos isolation kit (4478359/4484450, Thermo Fisher Scientific). For Exos isolation from CAFs/NFs supernatants, the samples were centrifuged at 300×g for 10 min to remove cells, and then centrifuged at 2000×*g* for 20 min to discard the cellular debris. After that, the samples were subjected to centrifugation at 10 000×*g* for 30 min to remove apoptotic bodies. Then, the supernatant was added with the total Exos isolation reagent at a ratio of 2:1, followed by incubation at 4°C overnight. Following centrifugation with 10 000×*g* for 1 h at 4°C, the Exos pellets were collected, resuspended in PBS and stored at −80°C. For plasma Exos isolation, peripheral blood was collected, followed by centrifugation at 2000×*g* for 20 min at 4°C to isolate the plasma. The plasma was mixed with the total Exos isolation reagent at a ratio of 3:1 and incubated at 4°C overnight. The mixture was then centrifuged at 10 000×*g* for 1 h at 4°C. The supernatant was discarded, and the pellet was collected from the bottom of the tube. Finally, the Exos pellets were resuspended in PBS and stored at −80°C until further use.

The Exo morphology was observed by transmission electron microscopy (TEM, HITACHI, Tokyo, Japan). The NS300 system (NanoSight, Malvern, UK) was used to evaluate the size distribution of Exos. The presence of Exo markers (CD81, CD9, CD63, and TSG101) and deficiency of GM130, Calnexin, Annexin V, and Albumin were validated by western blotting.

### 2.17 Exo uptake and treatment

The isolated Exos were tagged with PKH67 dye (MINI67, Sigma‐Aldrich, Saint Louis, USA) for 40 min. Afterwards, NSCLC cells were added with PKH67‐tagged Exos. After incubation for 24 h, the uptake of Exos by NSCLC cells was detected under fluorescence microscopy (Olympus). NSCLC cells were treated with 1 × 10^11^ particles/mL Exos in cell experiments.

### 2.18 Animal experiments

Male BALB/c nude mice (4–6 weeks old, 16–20 g) were provided by Hunan SJA Laboratory Animal Co., Ltd (Changsha, China). There were three batches of animal experiments (*n* = 6 per group). The first batch of experiments included four groups: sh‐NC, sh‐KLF13, vector, KLF13; the second batch of experiments included six groups: control, Exos, inhibitor NC‐Exos, miR‐3126‐5p inhibitor‐Exos, miR‐3126‐5p inhibitor‐Exos+sh‐NC, miR‐3126‐5p inhibitor‐Exos+sh‐KLF13; the third batch of experiments included four groups: control, Exos, Exos+vehicle, Exos+BKM120. The right flanks of mice received subcutaneous injection of 2 × 10^6^ A549 or NCI‐H1299 cells infected with lentiviruses carrying sh‐NC, sh‐KLF13, vector, or KLF13 in 100 µL of normal saline. Exos derived from CAFs (5 × 10^10^ particles/mouse in 100 µL PBS) from different groups were delivered into the mice via the tail vein once a week.^31^ Mice received BKM120 (40 mg/kg/d) by oral gavage for two weeks.^32^ An immunocompetent model was established in male C57BL/6N mice (4–6 weeks old, 16–20 g) obtained from Hunan SJA Laboratory Animal Co., Ltd. The mice were divided into control, Exos, Exos+vector, and Exos+KLF13 groups (*n* = 6 per group). LLC cells (1.5 × 10^5^ cells per mouse) infected with lentiviruses carrying vector or KLF13 were subcutaneously administered into the mice. The tumour volume was computed as follows: volume = length×width^2^/2. All mice underwent cervical dislocation for euthanasia at the end, and the xenografts were collected.

### 2.19 Immunohistochemical staining

The xenografts and clinical tissues were preserved in 4% paraformaldehyde, dehydrated via graded ethanol, and embedded before sectioning into 4 µm‐thick slices. The tissue sections were then dewaxed and rehydrated. Then, antigen repair was conducted in boiled citrate buffer, followed by blocking with 3% H_2_O_2_ for 10 min. Next, we incubated the sections with GLUT1 (ab150299, 1:200, Abcam), KLF13 (PA5‐99103, 1:200, Thermo Fisher Scientific), or Ki‐67 (ab15580, 1:100, Abcam) antibody overnight at 4°C and subsequent secondary antibody (ab6721, 1:1000, Abcam) for 2 h. Diaminobenzine (DAB) was used to visualize the sections that were then imaged under a light microscope (Olympus).

### 2.20 Reverse transcription quantitative polymerase chain reaction (RT‐qPCR)

Total RNA from tumours, cells, and Exos was purified via TRIzol (Thermo Fisher Scientific), then reverse‐transcribed to cDNA using the GoScript Reverse Transcription System (A5001, Promega). Then, qPCR was conducted on the 7500 Real‐Time PCR system (Applied Biosystems, Foster City, USA) utilizing the SYBR Premix Ex TaqTM II (RR820A, TaKaRa, Tokyo, Japan). Relative gene expression levels, normalized to β‐actin or U6, were determined using the 2^−ΔΔCt^ method.

### 2.21 Western blotting

We extracted protein samples using RIPA lysis buffer (Beyotime), quantified them via the BCA protein assay kit (PC0020, Solarbio), separated them by SDS‐PAGE, and blotted them onto polyvinylidene fluoride (PVDF) membranes. The PVDF membranes were then blocked with 5% skim milk for 1 h and treated with primary antibodies against KLF13 (PA5‐80754, 1:1000, Thermo Fisher Scientific), SH2B1 (12226‐1‐AP, 1:500, Proteintech), IRS1 (ab131487, 1:2000, Abcam), HK2 (22029‐1‐AP, 1:1500, Proteintech), GLUT1 (ab150299, 1:200, Abcam), PDK1 (ab202468, 1:2000, Abcam), ENO1 (ab227978, 1:1000, Abcam), LDHA (PA5‐27406, 1:3000, Thermo Fisher Scientific), p‐PI3K (PA5‐104853,1:500, Thermo Fisher Scientific), PI3K (PA5‐29220, 1:500, Thermo Fisher Scientific), p‐AKT (44‐621G, 1:1500, Thermo Fisher Scientific), AKT (MA5‐14916, 1:2000, Thermo Fisher Scientific), CD63 (ab134045, 1:1500, Abcam), TSG101 (ab125011, 1:2000, Abcam), CD9 (ab236630, 1:1500, Abcam), CD81 (ab155760, 1:1000, Abcam), GM130 (PA1‐077, 1:500, Thermo Fisher Scientific), Calnexin (ab22595, 1:1000, Abcam), Annexin V (ab108321, 1:1500, Abcam), Albumin (ab207327, 1:2000, Abcam), JAK2 (ab108596, 1:3000, Abcam), His (ab9108, 1:1000, Abcam), GST (ab111947, 1:1000, Abcam), and β‐actin (ab8227, 1:1000, Abcam) overnight at 4°C. After incubation with the secondary antibody (ab6721, 1:3000, Abcam) for 1.5 h, the bands were visualized using the West Pico ECL Substrate (PE0020, Solarbio).

### 2.22 Statistical analysis

Experiments were independently replicated at least three times. Data are shown as mean ± standard deviation (SD). Statistical analysis involved Student's *t*‐test for two‐group comparisons and one‐way analysis of variance (ANOVA) followed by Tukey's test for multiple groups using GraphPad Prism 8.0. Correlation between SH2B1 and KLF13/GLUT1 expression in NSCLC tissues and correlation between plasma exosomal miR‐3126‐5p level and GLUT1/LDHA expression in NSCLC tissues were tested by the Pearson correlation analysis. A *p*‐value less than.05 was considered statistically significant.

## 3 RESULTS

### 3.1 SH2B1 knockdown repressed glycolysis of NSCLC cells

**FIGURE 1:**
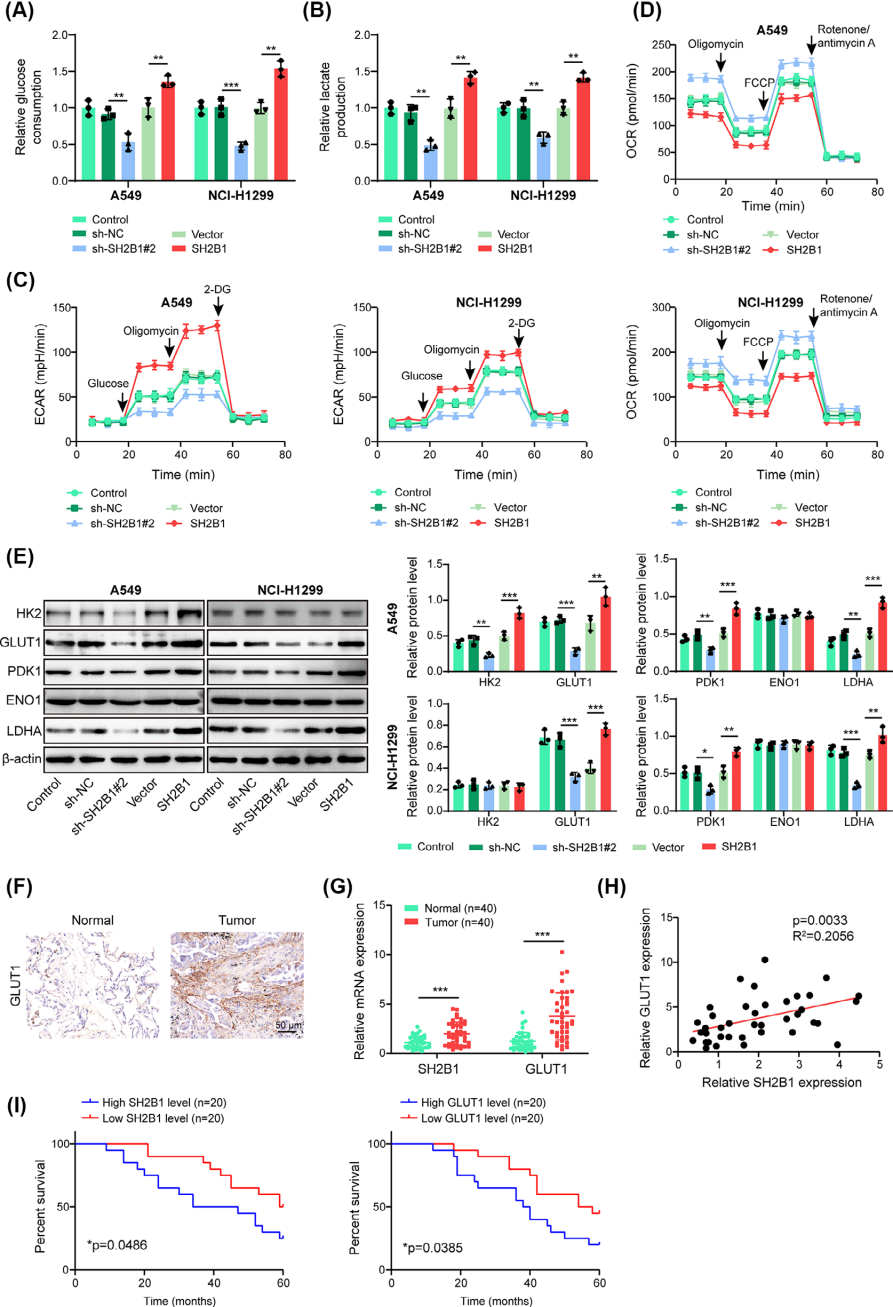
SH2B1 deficiency refrained glycolysis of NSCLC cells. NSCLC cells were transfected with sh‐SH2B1#2 or SH2B1 overexpression plasmid for 48 h. (A–D) Glucose uptake (A), lactate production (B), ECAR (C), and OCR (D) of NSCLC cells were detected by commercial kits, respectively. (E) Protein abundance of HK2, GLUT1, PDK1, ENO1, and LDHA was assessed by western blotting. (F) GLUT1 expression in NSCLC tissues and paired normal lung tissues was evaluated by immunohistochemical staining. Scale bar = 50 µm. (G) RT‐qPCR analysis of SH2B1 and GLUT1 mRNA levels in NSCLC and normal lung tissues (*n* = 40). (H) Correlation between SH2B1 and GLUT1 expression in NSCLC tissues was analyzed by the Pearson correlation analysis (*n* = 40). (I) The correlation between SH2B1/GLUT1 expression and survival of NSCLC patients was analyzed by Kaplan‐Meier plotter (*n* = 40). Student's *t*‐test (for G) or one‐way ANOVA followed by Tukey's test (for A–E) was adopted for statistical analysis. *p < .05, **p < .01, and ***p < .001.

To study the regulation of SH2B1 in the glycolysis of NSCLC, NSCLC cells were transfected with sh‐SH2B1#1‐3 or the SH2B1 overexpression plasmid. We found that SH2B1 was significantly knocked down by sh‐SH2B1#1‐2 or overexpressed after SH2B1 overexpression plasmid transfection (Figure S1A,B), and sh‐SH2B1#2, with the highest silencing efficiency, was selected in subsequent experiments. EdU staining (Figure S1C) and CCK‐8 assay (Figure S1D) indicated that the growth of NSCLC cells was restrained by SH2B1 knockdown, but enhanced by SH2B1 overexpression. In addition, SH2B1 silencing led to G_0_/G_1_ phase arrest, while SH2B1 overexpression exhibited the opposite roles (Figure S1E). Furthermore, SH2B1 depletion suppressed glucose consumption (Figure 1A) and lactate production (Figure 1B), reduced ECAR (Figure 1C), but raised OCR (Figure 1D) in NSCLC cells; however, we observed opposite results in the SH2B1‐overexpressed group (Figure 1A–D). Additionally, glycolysis markers including GLUT1, PDK1, and LDHA were downregulated by SH2B1 deficiency, while upregulated by SH2B1 overexpression (Figure 1E). Whereas, HK2 protein level in NCI‐H1299 cells and ENO1 protein level in both cells were not obviously changed among different groups (Figure 1E). Immunohistochemical staining showed a higher expression of GLUT1 in NSCLC tissues when compared with normal lung tissues (Figure 1F). Besides, SH2B1 and GLUT1 mRNA levels were elevated in NSCLC tissues (Figure 1G). A significant positive correlation was observed between SH2B1 and GLUT1 expression levels in NSCLC tissues (Figure 1H). Notably, high expression of SH2B1 or GLUT1 indicated a lower 5‐year survival of NSCLC patients (Figure 1I). Furthermore, we conducted experiments in SH2B1‐knockout cells. Western blotting validated the knockout efficiency in A549 and NCI‐H1299 cells (Figure S2A). Moreover, cell viability (Figure S2B), glucose uptake (Figure S2C), and lactate production (Figure S2D) were weakened in SH2B1‐knockout cells. Besides, SH2B1 knockout led to downregulation of GLUT1, PDK1, and LDHA in lung cancer cells (Figure S2E). Taken together, high expression of SH2B1 was correlated with higher glycolysis level, and SH2B1 downregulation suppressed glycolysis in NSCLC cells.

### 3.2 SH2B1 interacted with IRS1 to promote glycolysis in NSCLC cells

SH2B1 has been shown to drive the progression of lung adenocarcinoma via upregulation of IRS1.^20^ Thus, we further sought to validate the influence of the SH2B1/IRS1 axis on glycolysis of NSCLC cells. Co‐IP assay revealed an interaction between SH2B1 and IRS1 proteins, as well as between SH2B1 and positive control JAK2 (a known binding partner of SH2B1) in NSCLC cells (Figure S3A). Furthermore, the GST pull‐down assay demonstrated a direct interaction between SH2B1 and IRS1 proteins (Figure S3B). To explore the biological function of the SH2B1/IRS1 axis, the sh‐IRS1 and/or SH2B1 overexpression plasmid was transfected into NSCLC cells. Western blotting data showed that sh‐IRS1 transfection effectively reduced IRS1 protein level, but did not affect SH2B1 protein level. SH2B1 overexpression partly reversed sh‐IRS1‐mediated downregulation of IRS1 (Figure S3C). Functional experiments indicated that IRS1 silencing suppressed growth (Figure S3D,E) and caused G_0_/G_1_ phase arrest (Figure S3F) of NSCLC cells, which were partially counteracted by SH2B1 overexpression (Figure S3D–F). Besides, glucose consumption (Figure S4A), lactate production (Figure S4B), and ECAR (Figure S4C) were attenuated, but OCR (Figure S4D) was enhanced in IRS1‐depleted NSCLC cells, whereas these alterations were partly abolished by SH2B1 overexpression (Figure S4A–D). In addition, enforced expression of SH2B1 weakened IRS1 knockdown‐mediated downregulation of GLUT1, PDK1, LDHA, p‐PI3K, and p‐AKT in NSCLC cells (Figure S4E). These observations suggested that SH2B1 interplayed with IRS1 to promote glycolysis of NSCLC cells through activating the PI3K/AKT pathway.

### 3.3 KLF13 was downregulated in NSCLC and KLF13 overexpression repressed the transcription of SH2B1

**FIGURE 2:**
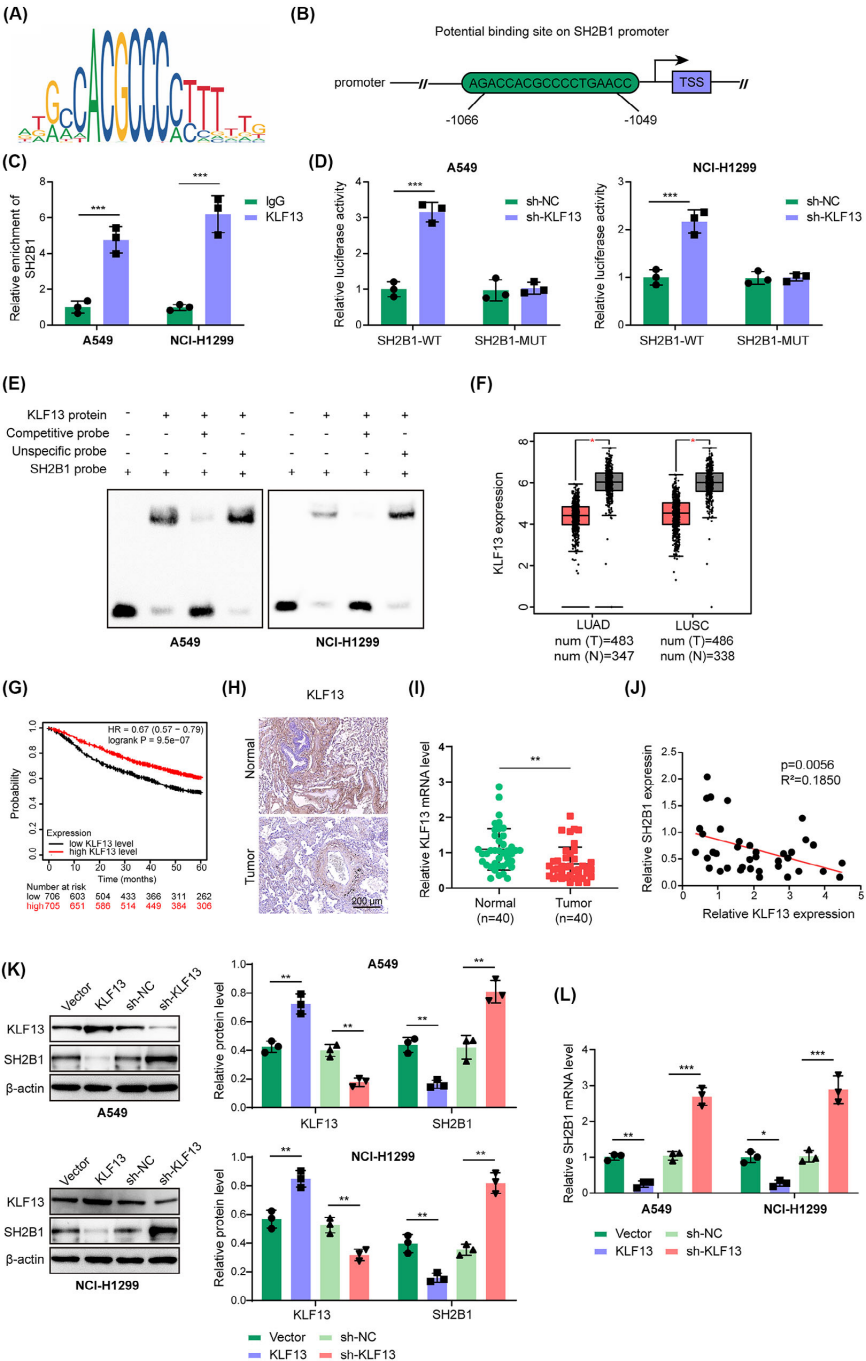
KLF13 bound to the SH2B1 promoter to repress its transcription in NSCLC cells. (A, B) JASPAR database (https://jaspar.elixir.no/) predicted the binding motif of KLF13 (A) and binding sites in promoter region of SH2B1 (B). (C–E) The direct binding of KLF13 to SH2B1 promoter was verified by ChIP (C), dual‐luciferase reporter assay (D), and EMSA (E). (F) GEPIA database (http://gepia.cancer‐pku.cn/) analyzed the differential expression of KLF13 in lung adenocarcinoma (LUAD) and lung squamous cell carcinoma (LUSC) tissues. For LUAD, number for tumour = 483; number for normal = 347. For LUSC, number for tumour = 486; number for normal = 338. (G) Kaplan–Meier Plotter database (https://kmplot.com/analysis/) evaluated the correlation between KLF13 expression and 5‐year overall survival of lung cancer patients (KLF13 low expression group, *n* = 706; KLF13 high expression group, *n* = 705). (H, I) KLF13 expression in NSCLC and normal lung tissues was detected by immunohistochemical staining (scale bar = 200 µm) (H) and RT‐qPCR (*n* = 40) (I). (J) Correlation between SH2B1 and KLF13 expression in NSCLC tissues was analyzed by the Pearson correlation analysis (*n* = 40). NSCLC cells were transfected with sh‐KLF13 or KLF13 overexpression plasmid for 48 h. (K) SH2B1 and KLF13 expression was detected by western blotting. (L) SH2B1 mRNA levels in NSCLC cells were tested by RT‐qPCR. Student's *t*‐test (for C, D, I) or one‐way ANOVA followed by Tukey's test (for K, L) was adopted for statistical analysis. *p < .05, **p < .01, and ***p < .001.

Next, the upstream core regulatory mechanism of SH2B1 in the glycolysis of NSCLC cells was elucidated. JASPAR database predicted that KLF13 could bind to the SH2B1 promoter region, and the binding motif of KLF13 (Figure 2A) and binding sites in the promoter region of SH2B1 (Figure 2B) were shown. ChIP assay proved that the SH2B1 promoter was evidently enriched with immunoprecipitation by KLF13 antibody (Figure 2C). Moreover, the relative luciferase activity of SH2B1‐WT was enhanced by KLF13 silencing, whereas the change disappeared in the SH2B1‐MUT group (Figure 2D). Furthermore, the EMSA assay demonstrated the direct binding of KLF13 protein to the SH2B1 promoter (Figure 2E). Our GEPIA database analysis revealed reduced KLF13 levels in lung squamous cell carcinoma (LUSC) and lung adenocarcinoma (LUAD) (Figure 2F). The lower KLF13 level was associated with a lower survival (Figure 2G). Consistently, KLF13 protein (Figure 2H) and mRNA (Figure 2I) levels were decreased in NSCLC samples. Moreover, KLF13 level was negatively associated with SH2B1 level in NSCLC tissues (Figure 2J). Lentiviruses‐mediated overexpression of KLF13 reduced SH2B1 expression; however, KLF13 deficiency remarkably raised SH2B1 expression (Figure 2K,L). Taken together, KLF13 directly bound to the SH2B1 promoter and restrained its transcription and expression in NSCLC cells.

### 3.4 KLF13 restrained glycolysis to delay NSCLC growth in vitro and in vivo

**FIGURE 3:**
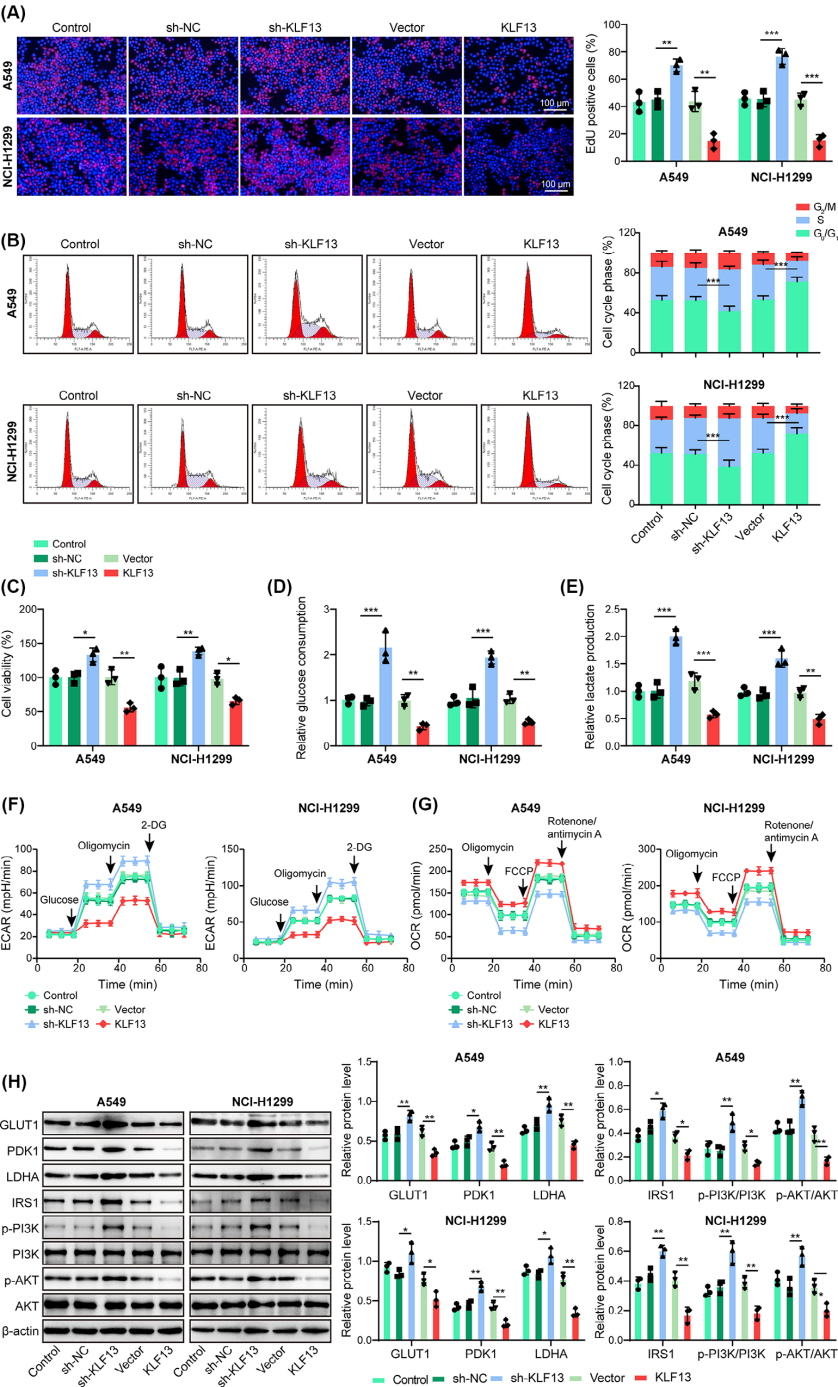
KLF13 inhibited glycolysis to suppress growth and cell cycle progression of NSCLC cells. NSCLC cells were transfected with sh‐KLF13 or KLF13 overexpression plasmid for 48 h. (A) EdU staining determined the proliferation of NSCLC cells. Scale bar = 100 µm. (B) Cell cycle progression of NSCLC cells was analyzed by PI staining combined with flow cytometry. (C) Cell viability of NSCLC cells was analyzed by CCK‐8 assay. (D–G) Glucose uptake (D), lactate production (E), ECAR (F), and OCR (G) of NSCLC cells were assessed by commercial kits, respectively. (H) Protein abundance of GLUT1, PDK1, LDHA, p‐PI3K, PI3K, p‐AKT, and AKT was measured by western blotting. ANOVA followed by Tukey's test was adopted for statistical analysis. *p < .05, **p < .01, and ***p < .001.

**FIGURE 4:**
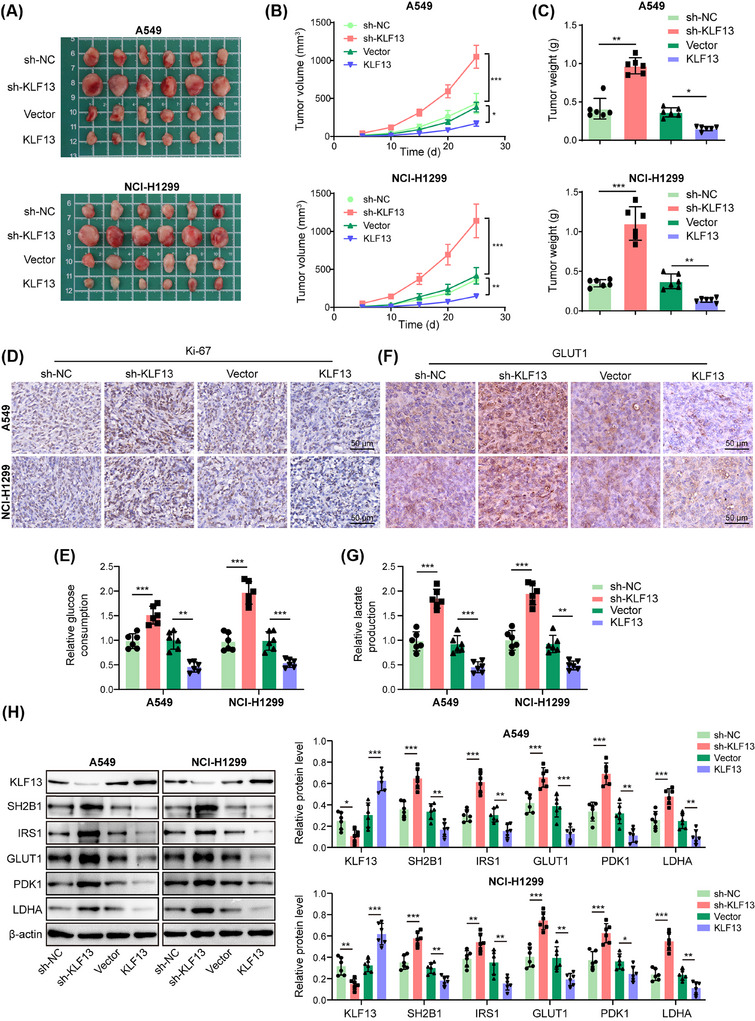
KLF13 inhibited glycolysis to delay NSCLC growth in vivo. The BALB/c nude mice were subcutaneously injected with A549 or NCI‐H1299 cells infected with lentiviruses carrying sh‐NC, sh‐KLF13, vector, or KLF13 overexpression plasmid. (A) The image for xenografts. (B) Tumour volume and (C) tumour weight were monitored. (D) Ki‐67 expression in tumours was evaluated by immunohistochemical staining (scale bar = 50 µm). (E) Glucose uptake in tumour tissues was measured using by commercial kit. (F) GLUT1 expression in tumours was analyzed by immunohistochemical staining (scale bar = 50 µm). (G) Lactate production in tumour tissues was measured using a commercial kit. (H) KLF13, SH2B1, IRS1, GLUT1, PDK1, and LDHA protein levels in tumours were assessed by western blotting. ANOVA followed by Tukey's test was adopted for statistical analysis. *p < .05, **p < .01, and ***p < .001.

Given that KLF13 acted as an upstream regulator of SH2B1, we further explored the function of KLF13 in the glycolysis of NSCLC cells. The cell growth was delayed (Figure 3A,C) and the G_0_/G_1_ phase was arrested (Figure 3B) in KLF13‐overexpressed NSCLC cells, and contrary results were found in the KLF13‐depleted group. Furthermore, KLF13 knockdown enhanced glucose consumption (Figure 3D), lactate production (Figure 3E), and ECAR (Figure 3F), but reduced OCR (Figure 3G) in NSCLC cells; however, KLF13 overexpression exerted the opposite roles (Figure 3D–G). Besides, GLUT1, PDK1, LDHA, IRS1, p‐PI3K, and p‐AKT were upregulated by KLF13 silencing, but downregulated by KLF13 overexpression (Figure 3H). Therefore, KLF13 exerted an inhibitory effect on the glycolysis of NSCLC cells. To corroborate the in vitro experimental results, we developed a mouse xenograft model. KLF13 silencing increased tumour volume and weight, whereas its overexpression reduced these metrics (Figure 4A‐C). In addition, KLF13 ablation enhanced Ki‐67 expression in the xenografts, whereas KLF13 overexpression decreased Ki‐67 expression (Figure 4D). As expected, glucose consumption (Figure 4E), lactate production (Figure 4G), and GLUT1 expression (Figure 4F) were intensified by KLF13 downregulation but weakened by KLF13 overexpression. Additionally, silencing of KLF13 raised the protein levels of SH2B1, IRS1, GLUT1, PDK1, and LDHA in xenograft tumours, and KLF13 overexpression exhibited the reverse actions (Figure 4H). The above data indicated that KLF13 repressed glycolysis to delay NSCLC progression in vitro and in vivo.

### 3.5 KLF13 suppressed the glycolysis of NSCLC cells via downregulation of SH2B1

To explore the regulation of the KLF13/SH2B1 axis in glycolysis, NSCLC cells were transfected with KLF13 and SH2B1 overexpression plasmids. KLF13 overexpression strikingly reduced SH2B1, IRS1, p‐PI3K, and p‐AKT protein levels, which were restored by SH2B1 overexpression (Figure S5A). Furthermore, KLF13 overexpression‐mediated G_0_/G_1_ phase arrest and inhibition of cell proliferation were neutralized in SH2B1‐overexpressed NSCLC cells (Figure S5B,C). In addition, the attenuated glucose consumption (Figure S5D), lactate production (Figure S5E), ECAR (Figure S5F), and enhanced OCR (Figure S5G) in the KLF13 overexpression group were reversed by co‐overexpression of SH2B1. Accordingly, downregulation of GLUT1, PDK1, and LDHA mediated by KLF13 overexpression was recovered when SH2B1 was overexpressed (Figure S5H). To sum up, KLF13 inhibited SH2B1 expression to repress the glycolysis of NSCLC cells.

### 3.6 CAFs‐derived Exos repressed KLF13 expression in NSCLC cells

**FIGURE 5:**
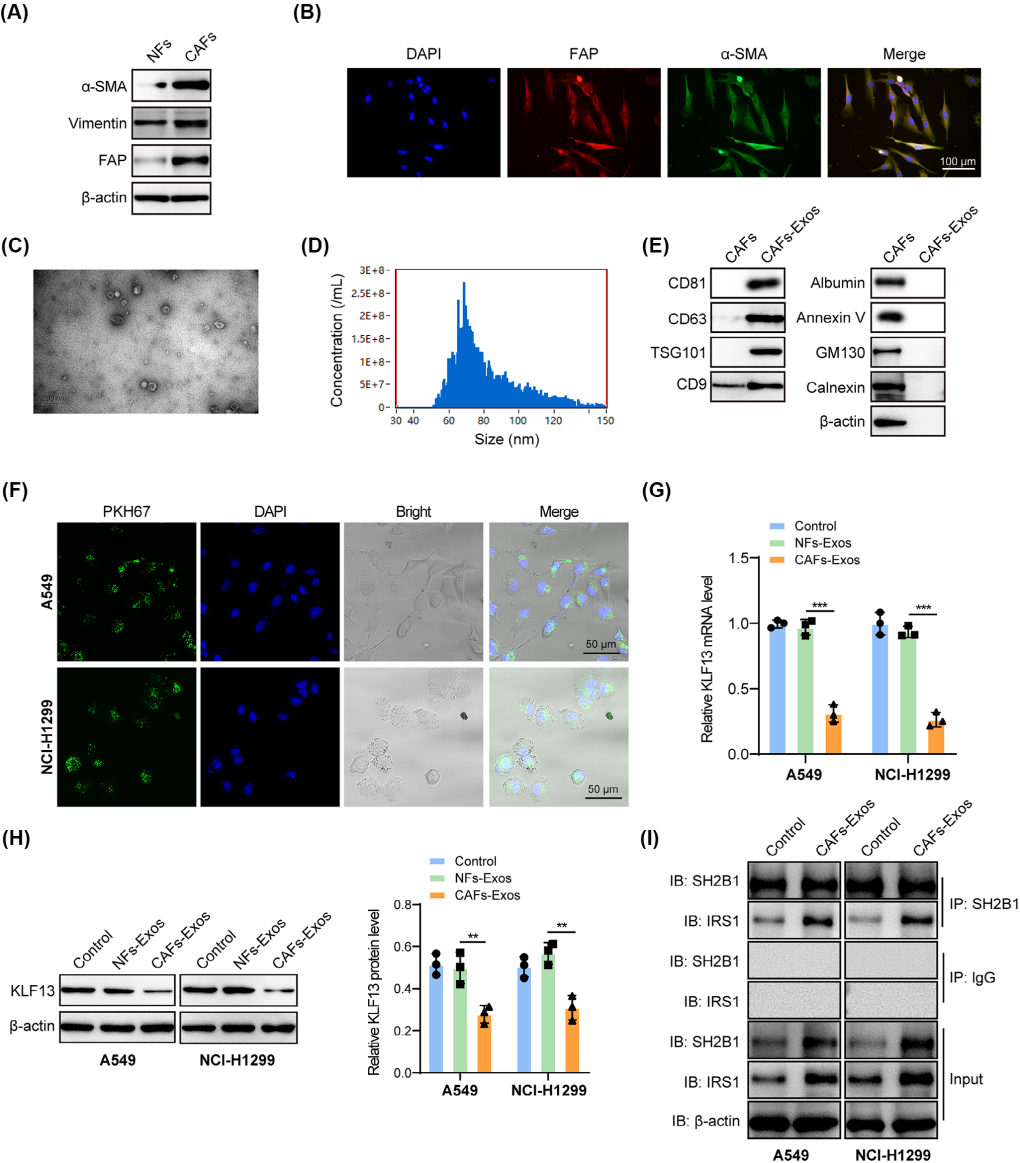
CAFs‐derived Exos led to a reduction of KLF13 expression in NSCLC cells. (A) Western blotting and (B) immunofluorescence staining (scale bar = 100 µm) evaluated the expression of α‐SMA, vimentin, and FAP in isolated CAFs and NFs. (C) The morphology of CAFs‐derived Exos was observed by TEM. Scale bar = 200 nm. (D) The size distribution of Exos was determined by nanoparticle tracking analysis. (E) The protein levels of CD9, CD63, TSG101, CD81, GM130, Calnexin, Annexin V, and Albumin in CAFs and CAFs‐derived Exos were analyzed by western blotting. (F) The uptake of PKH67‐labelled Exos by NSCLC cells was observed under a fluorescence microscope (scale bar = 50 µm). (G, H) NSCLC cells were treated with NFs/CAFs‐derived Exos, and KLF13 expression was detected by RT‐qPCR (G) and western blotting (H). (I) Co‐IP assay was performed to examine the effect of CAFs‐derived Exos on the interaction between SH2B1 and IRS1 proteins in NSCLC cells. NSCLC cells were treated with 1×1011 particles/mL Exos in cell experiments. Student's *t*‐test (for A, E) or one‐way ANOVA followed by Tukey's test (for G, H) was adopted for statistical analysis. **p < .01 and ***p < .001.

To elucidate the possible mechanism responsible for the low expression of KLF13 in NSCLC cells, we focused on CAFs, a major constituent in the tumour microenvironment. CAFs and NFs were extracted from NSCLC samples and normal tissues. As shown in Figure 5A,B, CAFs possessed higher expression of α‐SMA, vimentin, and FAP as compared with NFs. Exos derived from CAFs have been recognized as messengers between CAFs and tumour cells.^33^ Thus, Exos were isolated from CAFs, and TEM showed that Exos exhibited as round or oval membranous vesicles with a diameter of about 100 nm (Figure 5C). Nanoparticle tracking analysis indicated that Exos showed a typical size distribution from 50 to 150 nm (Figure 5D). Western blotting further validated the presence of Exos markers CD9, CD63, TSG101, and CD81, while the absence of GM130 (Golgi apparatus marker), Calnexin (endoplasmic reticulum marker), Annexin V (microvesicle marker), and Albumin (soluble protein marker) in Exos (Figure 5E). Moreover, the accumulated green fluorescence of PKH67 suggested the efficient uptake of Exos by NSCLC cells (Figure 5F). Of note, KLF13 expression was evidently reduced after treatment with CAFs‐derived Exos, but not affected by NFs‐derived Exos (Figure 5G,H). Co‐IP assay showed that CAFs‐derived Exos treatment not only enhanced the expression of SH2B1 and IRS1, but also promoted their interaction (Figure 5I). These findings revealed that CAFs‐derived Exos were responsible for the decreased expression of KLF13 in NSCLC cells.

### 3.7 Exosomal miR‐3126‐5p from CAFs targeted KLF13 in NSCLC cells

**FIGURE 6:**
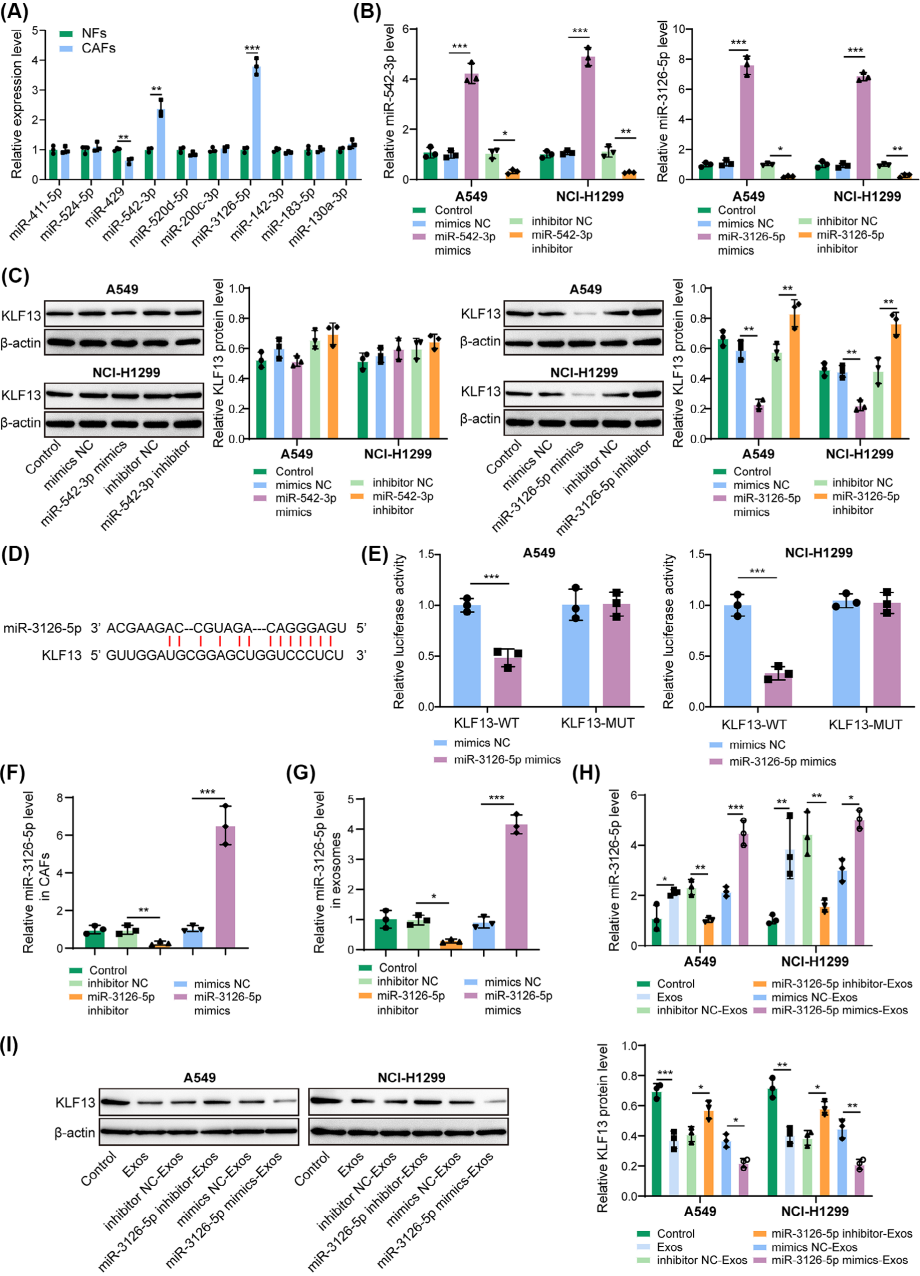
CAFs‐derived Exos delivered miR‐3126‐5p to inhibit KLF13 expression in NSCLC cells. (A) ENCORI database (https://rnasysu.com/encori/) was adopted to predict the miRNAs possessing binding sites to KLF13 3′‐UTR. RT‐qPCR analyses of differential expression of top 10 miRNAs (miR‐411‐5p, miR‐524‐5p, miR‐429, miR‐542‐3p, miR‐520d‐5p, miR‐200c‐3p, miR‐3126‐5p, miR‐142‐3p, miR‐183‐5p, and miR‐130a‐3p) with potential binding sites to 3′‐UTR of KLF13 mRNA in NFs and CAFs. NSCLC cells were transfected with miR‐542‐3p mimics/inhibitor, miR‐3126‐5p mimics/inhibitor, or mimics/inhibitor NC for 48 h. (B) MiR‐542‐3p and miR‐3126‐5p expression in NSCLC cells was assessed by RT‐qPCR. (C) KLF13 protein abundance was determined by western blotting. (D) The binding sites of miR‐3126‐5p to the 3′‐UTR of KLF13 mRNA were predicted by the ENCORI database (https://rnasysu.com/encori/). (E) The binding of miR‐3126‐5p to the 3′‐UTR of KLF13 mRNA was validated by dual‐luciferase reporter assay. (F, G) CAFs were transfected with miR‐3126‐5p mimics/inhibitor, or mimics/inhibitor NC. The expression of miR‐3126‐5p in CAFs (F) and CAFs‐derived Exos (G) was detected by RT‐qPCR. NSCLC cells were treated with Exos (1 × 10^11^ particles/mL) isolated from CAFs transfected with miR‐3126‐5p mimics/inhibitor, or mimics/inhibitor NC. (H) MiR‐3126‐5p expression in NSCLC cells was assessed by RT‐qPCR. (I) KLF13 protein levels in NSCLC cells were analyzed by western blotting. Student's *t*‐test (for A, E) or one‐way ANOVA followed by Tukey's test (for B, C, F–I) was adopted for statistical analysis. *p < .05, **p < .01, and ***p < .001.

To clarify the precise mechanism underlying KLF13 inhibition in NSCLC cells mediated by CAFs‐derived Exos, we detected the differential expression of the top 10 miRNAs with potential binding sites to KLF13 3′‐UTR in NFs and CAFs. According to the results, miR‐542‐3p and miR‐3126‐5p levels were significantly increased in CAFs than in NFs (Figure 6A). Furthermore, overexpression or inhibition of miR‐542‐3p did not affect KLF13 expression in NSCLC cells (Figure 6B,C). Notably, miR‐3126‐5p overexpression led to a significant downregulation of KLF13 expression, whereas its inhibition resulted in marked upregulation (Figure 6B,C). Based on these results, miR‐3126‐5p was selected in the following experiments. The binding sites of miR‐3126‐5p to KLF13 mRNA 3′‐UTR were illustrated in Figure 6D. Besides, miR‐3126‐5p mimics evidently lowered the relative luciferase activity of KLF13‐WT, rather than KLF13‐MUT (Figure 6E). Additionally, miR‐3126‐5p inhibitor transfection remarkably reduced miR‐3126‐5p level in CAFs and their Exos, whereas miR‐3126‐5p mimics led to elevation in miR‐3126‐5p expression in CAFs and their Exos (Figure 6F,G). Subsequently, NSCLC cells were treated with Exos derived from miR‐3126‐5p‐silenced/overexpressed CAFs. We found that treatment with miR‐3126‐5p inhibitor‐Exos restrained miR‐3126‐5p expression (Figure 6H) and raised KLF13 expression (Figure 6I) in NSCLC cells, and these results were reversed in the miR‐3126‐5p mimics‐Exos group (Figure 6H,I). Therefore, miR‐3126‐5p was shuttled from CAFs‐derived Exos to NSCLC cells, and thus targeting KLF13 to inhibit its expression.

### 3.8 Exosomal miR‐3126‐5p contributed to glycolysis of NSCLC cells via targeting KLF13

**FIGURE 7:**
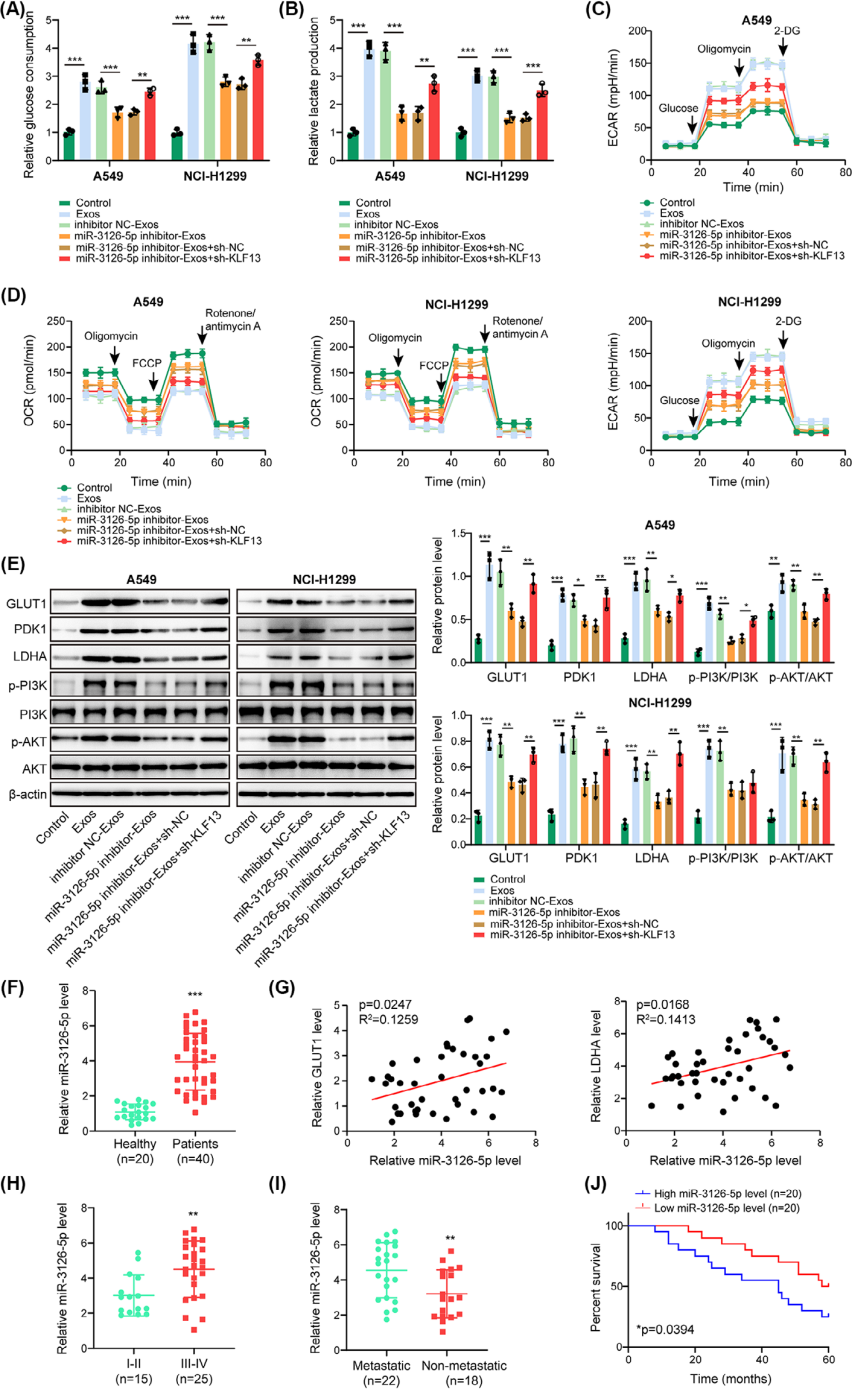
Exosomal miR‐3126‐5p targeted KLF13 to facilitate glycolysis of NSCLC cells. NSCLC cells were treated with Exos (1 × 10^11^ particles/mL) isolated from CAFs from different groups, combined with or without sh‐KLF13 transfection. (A–D) Glucose uptake (A), lactate production (B), ECAR (C), and OCR (D) of NSCLC cells were measured by commercial kits, respectively. (E) GLUT1, PDK1, LDHA, p‐PI3K, PI3K, p‐AKT and AKT protein levels were analyzed by western blotting. (F) Exosomal miR‐3126‐5p in the peripheral blood plasma of healthy volunteers (*n* = 20) and NSCLC patients (*n* = 40) was detected by RT‐qPCR. (G) Correlations between exosomal miR‐3126‐5p in plasma and GLUT1/LDHA expression in NSCLC tissues were analyzed by Pearson correlation analysis (*n* = 40). (H) RT‐qPCR analysis of exosomal miR‐3126‐5p in the peripheral blood plasma of NSCLC patients in stage I‐II (*n* = 15) and stage III‐IV (*n* = 25). (I) Exosomal miR‐3126‐5p level in the peripheral blood plasma of NSCLC patients with metastasis (*n* = 22) and non‐metastasis (*n* = 18) was assessed by RT‐qPCR. (J) The correlation between plasma exosomal miR‐3126‐5p expression and survival of NSCLC patients was analyzed by Kaplan–Meier plotter (*n* = 40). Student's *t*‐test (for F, H, I) or one‐way ANOVA followed by Tukey's test (for A–E) was adopted for statistical analysis. *p < .05, **p < .01, and ***p < .001.

Next, we sought to examine the role of the exosomal miR‐3126‐5p/KLF13 axis in the glycolysis of NSCLC cells. Treatment with Exos strikingly raised miR‐3126‐5p expression in NSCLC cells, which was abolished by miR‐3126‐5p inhibitor, but not affected by sh‐KLF13 transfection (Figure S6A). Knockdown of exosomal miR‐3126‐5p derived from CAFs reversed the downregulation of KLF13 and upregulation of SH2B1 and IRS1 in Exos‐treated NSCLC cells, and these changes were abrogated by KLF13 knockdown in NSCLC cells (Figure S6B). The cell cycle progression (Figure S6C,D) and cell proliferation (Figure S6E,F) were promoted by Exos derived from CAFs, which were weakened by miR‐3126‐5p‐silenced Exos, whereas the suppressive effect of miR‐3126‐5p‐depleted Exos on cell proliferation and cell cycle progression was neutralized by the deficiency of KLF13 in NSCLC cells (Figure S6C–F). Additionally, the enhanced levels of glucose consumption (Figure 7A), lactate production (Figure 7B), ECAR (Figure 7C), GLUT1, PDK1, LDHA, p‐PI3K, p‐AKT (Figure 7E), and reduced level of OCR (Figure 7D) in Exos‐treated NSCLC cells were partially reversed by depletion of miR‐3126‐5p. However, these changes mediated by miR‐3126‐5p‐silenced Exos were abolished by KLF13 knockdown in NSCLC cells (Figure 7A–E). Furthermore, exosomal miR‐3126‐5p level was higher in the peripheral blood plasma of NSCLC patients as compared with healthy volunteers (Figure 7F). Besides, exosomal miR‐3126‐5p level in plasma was positively associated with GLUT1/LDHA expression in NSCLC tissues (Figure 7G). Moreover, plasma exosomal miR‐3126‐5p level was higher in stage III‐IV than that in stage I‐II of NSCLC patients (Figure 7H). A higher exosomal miR‐3126‐5p level in plasma was also found in NSCLC patients with metastasis compared with non‐metastasis (Figure 7I). Notably, high exosomal miR‐3126‐5p level in plasma was correlated with a low survival of NSCLC patients (Figure 7J). The above observations revealed that exosomal miR‐3126‐5p acted as a driver of glycolysis in NSCLC cells via inhibition of KLF13 expression.

### 3.9 Exosomal miR‐3126‐5p favoured NSCLC growth in vivo by repressing KLF13 expression to trigger glycolysis

**FIGURE 8:**
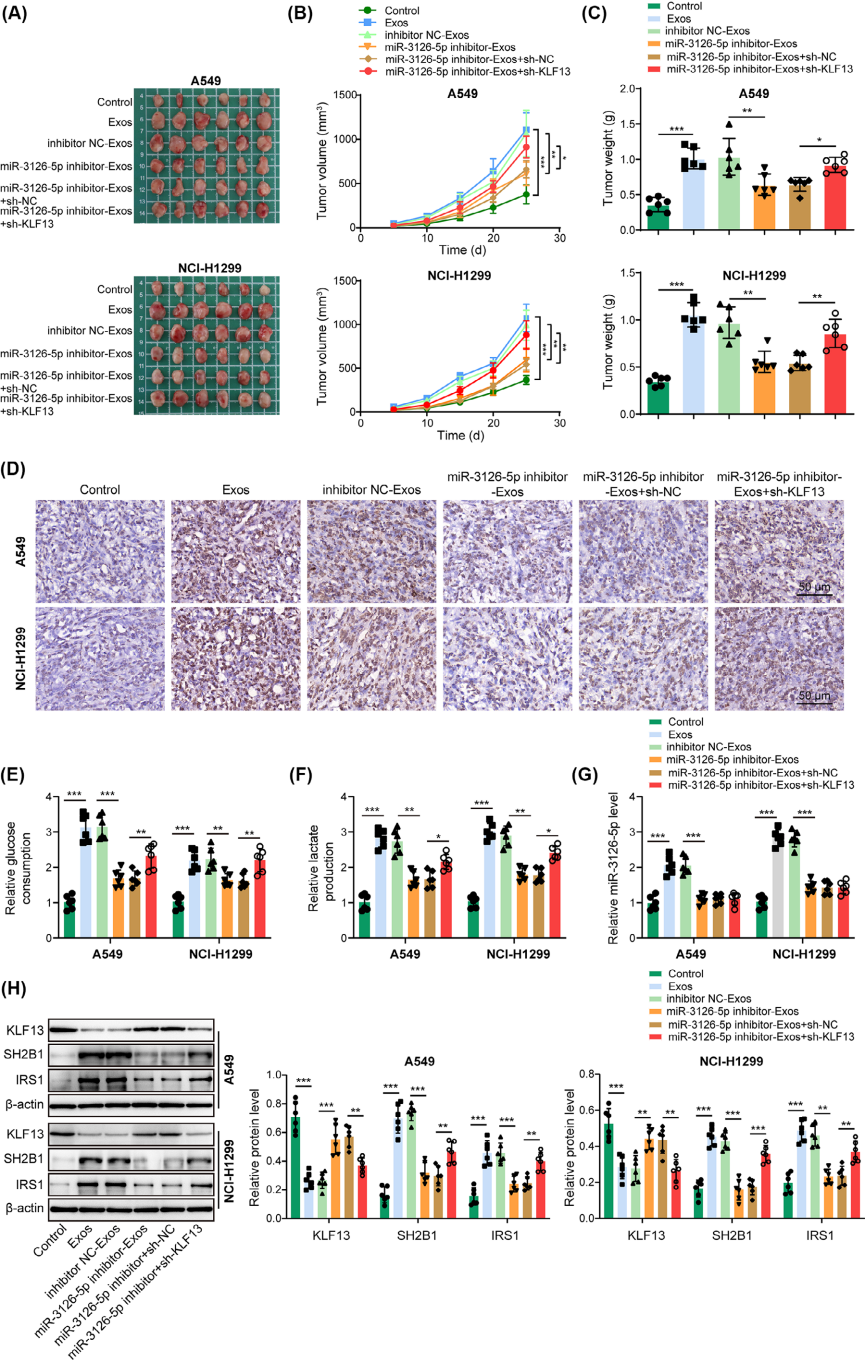
Exosomal miR‐3126‐5p triggered glycolysis to accelerate NSCLC growth in vivo by downregulation of KLF13. The BALB/c nude mice were subcutaneously injected with A549 or NCI‐H1299 cells infected with lentiviruses carrying sh‐NC or sh‐KLF13 in combination with tail vein injection of Exos (5 × 10^10^ particles/mouse in 100 µL PBS) derived from CAFs from different groups once a week. (A) The image for xenografts. (B) Tumour volume and (C) tumour weight were monitored. (D) Ki‐67 expression in tumours was observed by immunohistochemical staining (scale bar = 50 µm). (E‐F) Glucose uptake (E) and lactate production (F) in tumour tissues were measured by commercial kits. (G) MiR‐3126‐5p expression in tumours was analyzed by RT‐qPCR. (H) KLF13, SH2B1, and IRS1 protein levels in tumours were assessed by western blotting. ANOVA followed by Tukey's test was adopted for statistical analysis. *p < .05, **p < .01, and ***p < .001.

Finally, we validated the in vitro findings of the exosomal miR‐3126‐5p/KLF13 axis in a xenograft mouse model. As illustrated in Figure 8A–D, CAFs‐derived Exos promoted tumour growth and Ki‐67 expression in tumours. Treatment with miR‐3126‐5p‐silenced Exos remarkably restrained tumour growth (Figure 8A–C) and Ki‐67 expression (Figure 8D), which were restored by KLF13 deficiency. Furthermore, the elevated glucose consumption (Figure 8E) and lactate production (Figure 8F) in tumours of the Exos treatment group were reversed by miR‐3126‐5p deficiency, which were restored by KLF13 knockdown. Accordingly, Exos treatment‐mediated elevation in miR‐3126‐5p, SH2B1, IRS1 expression, and reduction in KLF13 expression were reversed by miR‐3126‐5p‐silenced Exos (Figure 8G,H). However, KLF13 downregulation counteracted these changes except change in miR‐3126‐5p expression (Figure 8G,H). Additionally, we developed an immunocompetent mouse model by injecting C57BL/6N mice with murine Lewis lung carcinoma LLC cells. We found that treatment with Exos significantly facilitated tumour growth (Figure S7A–C), glucose consumption (Figure S7D) and lactate production (Figure S7E) in tumours, which were abolished by KLF13 overexpression. Furthermore, miR‐3126‐5p, SH2B1, and IRS1 levels were elevated, but KLF13 level was reduced in tumours of Exos‐treated mice, whereas KLF13 overexpression counteracted these changes, except change in miR‐3126‐5p expression (Figure S7F,G). In order to verify the role of the PI3K/AKT signalling pathway in this process, an inhibitor of this pathway, BKM120, was applied. CAFs‐derived Exos‐mediated promotion in tumour growth (Figure S8A–C), glucose consumption (Figure S8D) and lactate production (Figure S8E) in tumours were weakened by combination treatment with BKM120 in BALB/c nude mice. In addition, upregulation of p‐AKT in Exos‐treated tumours was reversed by BKM120, whereas downregulation of KLF13 and upregulation of miR‐3126‐5p, SH2B1, IRS1, and p‐PI3K caused by Exos were not affected by BKM120 co‐administration (Figure S8F,G). Collectively, CAFs‐derived exosomal miR‐3126‐5p facilitated glycolysis to accelerate in vivo NSCLC growth through repressing KLF13 expression and the PI3K/AKT pathway.

## 4 DISCUSSION

Lack of discernible clinical manifestations and diagnostic biomarkers remains a huge challenge for NSCLC management, resulting in a poor prognosis for most patients due to late diagnosis.^34^ Tumour cells exhibit a remarkable metabolic phenotype, featured by elevated glycolysis.^35^ This glucose metabolic reprogramming supports the malignant development of NSCLC.^36^ CAFs are considered a key component in the tumour microenvironment, which has been demonstrated to affect metabolic reprogramming of lung cancer.^37^, ^38^ In this work, we discovered that a higher exosomal miR‐3126‐5p level in peripheral blood plasma suggested a poor prognosis of NSCLC patients, and CAFs‐derived exosomal miR‐3126‐5p targeted KLF13 and consequently activated the SH2B1/IRS1 axis to promote PI3K/AKT pathway‐mediated glycolysis, thus accelerating the progression of NSCLC. These insights indicate that inhibition of CAFs‐derived exosomal miR‐3126‐5p in the tumour microenvironment holds promise as an effective therapeutic target for inhibiting glucose metabolic reprogramming in NSCLC.

SH2B1, an adaptor protein, has been widely recognized to be an oncogene in various malignancies. For instance, SH2B1 exhibited higher expression in colorectal cancer, and this was correlated with the adverse prognosis.^39^ Moreover, SH2B1 was upregulated by circ_0075960 via sponging miR‐361‐3p, which facilitated endometrial carcinoma development.^40^ Besides serving as an oncogene, SH2B1 also exerts crucial roles in energy homeostasis, obesity, and glucose metabolism.^16^ Ablation of SH2B1 resulted in energy imbalance, obesity, and metabolic dysfunction via repressing BDNF action in mice.^41^ SH2B1 silencing was documented to reduce glycolysis in a cardiac hypertrophy model.^42^ However, the regulation of SH2B1 in the glycolysis of NSCLC cells remains largely unknown. Herein, we first proved that SH2B1 was positively associated with GLUT1 level in NSCLC samples. Furthermore, SH2B1 overexpression enhanced glucose uptake, lactate production, ECAR, reduced OCR, and upregulated glycolysis markers GLUT1, PDK1, and LDHA in NSCLC cells, suggesting the promoting roles in glycolysis of NSCLC. Of note, SH2B1 did not affect the expression of ENO1 in both A549 and NCI‐H1299 cells, indicating that the ENO1 expression in NSCLC might be regulated via other factors and mechanisms. Besides, the HK2 protein level in NCI‐H1299 cells was not influenced by SH2B1. HK2 is a key rate‐limiting enzyme in glycolysis, with its expression directly and positively correlating with glycolytic flow. The possible reason for the differential expression of HK2 in various NSCLC cells might be the different “glycolytic types” of NSCLC cells. Mounting evidence proved that IRS1, a key component of the insulin receptor substrate family, was involved in the regulation of lipid metabolism.^43^, ^44^, ^45^ Besides the regulation in lipid metabolism, IRS1 was validated as a key regulator of glucose metabolism and insulin resistance.^46^ Previous studies found that IRS1 was involved in the malignant transformation of cancers, which exerted a pivotal role in the progression of NSCLC.^47^, ^48^ However, investigation of IRS1 in glycolysis during NSCLC is still lacking. It was demonstrated that SH2B1 interacted with IRS1 in various pathophysiological processes. For example, SH2B1 directly interacted with IRS1 in response to leptin stimulation.^49^ Consistent with the above observations, we first confirmed that SH2B1 directly interacted with IRS1 to drive glycolysis of NSCLC cells via activating the PI3K/AKT pathway. These findings suggested that IRS1 was a downstream target for SH2B1 in the modulation of glycolysis during NSCLC progression.

This study further gained insight into the upstream mechanism of the SH2B1/IRS1 axis in the glycolysis of NSCLC. Specifically, the modulation of gene expression via transcription factors has become a pivotal determinant of dysregulation of genes in NSCLC.^50^ KLF13 is one of the important transcription factors which can bind to GC‐rich sequences.^51^ Recent studies have revealed that KLF13 exhibits dual roles as a tumour suppressor or oncogenic driver across diverse malignancies. For example, KLF13 suppressed colorectal cancer development via repressing HMGCS1 transcription.^28^ On the contrary, KLF13 facilitated hepatocellular carcinoma progression via transcriptional activation of Acyl‐CoA thioesterase 7 to promote fatty acid metabolism.^52^ For lung adenocarcinoma, KLF13 induced ferroptosis via repressing transcription of GPX4, thus sensitizing lung adenocarcinoma cells to chemotherapy drugs.^53^ The other work documented that KLF13 restrained epithelial–mesenchymal transition of lung adenocarcinoma cells through inhibiting TROAP transcription.^54^ But whether KLF13 can modulate NSCLC glycolysis has not been clarified. Our data uncovered that KLF13 directly bound to the SH2B1 promoter to restrain its transcription and expression, thereby inhibiting glycolysis to delay progression of NSCLC in vitro and in vivo. Our study first revealed the transcriptional inhibition of SH2B1 by KLF13 in NSCLC.

CAFs represent the most plentiful components in the tumour microenvironment, which can affect key processes of cancers, such as metastasis and glycolysis.^14^ It has been indicated that miRNAs can be loaded by Exos and then transferred to recipient cells, where miRNAs post‐transcriptionally suppress target gene expression through recognizing and binding to the 3′‐UTR.^55^ In a previous study, miR‐200 was secreted by CAFs via Exos, which repressed metastasis of NSCLC cells via targeting ZEB1.^56^ Sun et al.^57^ discovered that CAFs‐derived exosomal miR‐3124‐5p conferred malignant development of NSCLC cells by targeting the TOLLIP/TLR4/MyD88/NF‐κB axis. Another study reported that CAFs‐secreted midkine contributed to the glycolysis of NSCLC cells via activating the Wnt/β‐catenin pathway.^58^ Herein, we discovered that miR‐3126‐5p was upregulated in CAFs‐derived Exos, which were delivered to NSCLC cells and facilitated glycolysis via transcriptional inhibition of KLF13. Besides, circulating exosomal miR‐3126‐5p in plasma was also increased in NSCLC patients, with a positive correlation with GLUT1/LDHA level and low survival in NSCLC patients, which provided a clinical interrelation with glucose metabolic reprogramming. Therefore, plasma circulating exosomal miR‐3126‐5p levels may serve as an early diagnostic biomarker for NSCLC. In vivo data demonstrated that CAFs‐derived exosomal miR‐3126‐5p facilitated NSCLC progression by targeting KLF13 in both immunodeficient nude mice and immunocompetent C57BL/6N mice. Moreover, the promotion in NSCLC growth and glycolysis mediated by CAFs‐derived Exos in vivo was reversed by PI3K/AKT pathway inhibitor BKM120, proving the involvement of the PI3K/AKT pathway in this process. Of note, our results proved that miR‐542‐3p was also highly enriched in CAFs‐derived Exos. However, KLF13 was not the downstream target of CAFs‐derived exosomal miR‐542‐3p. In this study, we mainly focused on exogenous regulation of KLF13 expression by CAFs‐derived exosomal miR‐3126‐5p. However, other endogenous mechanisms might also be responsible for the low expression of KLF13 in NSCLS (such as epigenetic modification, promoter site competition, and endogenous miRNA regulation).^59^, ^60^, ^61^, ^62^ We will investigate whether KLF13 can be modulated by these endogenous mechanisms in the future. This is the first report demonstrating the contribution of CAFs‐derived exosomal miR‐3126‐5p/KLF13 axis to glycolysis in NSCLC cells.

There are several limitations. First, the biological roles of other exosomal miRNAs, such as miR‐542‐3p in NSCLC and related mechanisms, remain unelucidated. Due to the limitations of time and research funds, we were unable to conduct a functional exploration of miR‐542‐3p in this study. But there is no report about the role of miR‐542‐3p in NSCLC glycolysis by searching the existing literature, indicating that miR‐542‐3p might exert its role via other mechanisms apart from the KLF13/SH2B1 axis and glycolysis. Second, this study provided extensive evidence to prove the regulation of the exosomal miR‐3126‐5p/KLF13/SH2B1 pathway in the glycolysis of NSCLC. We cannot rule out the possibility that other regulatory mechanisms, such as epigenetic modifications or transcription factor competition,^63^, ^64^, ^65^ may also be involved in the regulation of SH2B1. These issues will be important directions for future research.

## 5 CONCLUSION

Taken together, we uncovered a new modulatory mechanism of glycolysis in NSCLC. CAFs‐derived Exos delivered miR‐3126‐5p to downregulate KLF13 in NSCLC cells. Moreover, the decreased expression of KLF13 led to transcriptional activation of SH2B1, which interacted with IRS1 to facilitate PI3K/AKT pathway‐mediated glycolysis of NSCLC cells. Our results shed novel light on the treatment potential of targeting CAFs‐derived exosomal miR‐3126‐5p to repress glycolysis and improve the prognosis of NSCLC patients.

## AUTHOR CONTRIBUTIONS

**Zhenyu Zhang**: Conceptualization; data curation; writing–original draft; writing–review & editing. **Haicheng Ma**: Data curation; investigation. **Yingying Zheng**: Project administration; supervision. **Lina Wang**: Funding acquisition; resources. **Chenghui Wang**: Methodology; software. **Yuanyuan Liu**: Supervision; visualization. **Hengxiao Lu**: Supervision; validation; visualization. **Shaoqiang Wang**: Funding acquisition; resources; software, supervision; writing–review & editing.

## CONFLICT OF INTEREST STATEMENT

The authors declare no conflict of interest.

## ETHICS STATEMENT

Ethical permission was granted by the Ethics Committee of the Affiliated Hospital of Jining Medical University (Ethical number: 2022B033). The animal experimental protocol was approved by the Animal Care and Use Committee of the Affiliated Hospital of Jining Medical University.

## Supporting information




**Figure S1 SH2B1 deficiency restrained growth and cell cycle progression of NSCLC cells**. (A, B) NSCLC cells were transfected with sh‐SH2B1#1, 2, 3 or SH2B1 overexpression plasmid for 48 h, and RT‐qPCR (A) and western blotting (B) analyses of SH2B1 expression. NSCLC cells were transfected with sh‐SH2B1#2 or SH2B1 overexpression plasmid for 48 h. (C, D) The growth of NSCLC cells was evaluated by EdU staining (C) and CCK‐8 assay (D). Scale bar = 100 µm in C. (E) Cell cycle progression was determined by PI staining and flow cytometry. ANOVA followed by Tukey's test was adopted for statistical analysis. **p < .01 and ***p < .001.
**Figure S2 SH2B1 knockout repressed the glycolysis of NSCLC cells**. (A) Western blotting analysis of SH2B1 protein level in parental and SH2B1‐knockout NSCLC cells. (B) Cell viability was determined by CCK‐8. (C, D) Glucose uptake (C) and lactate production (D) were detected by commercial kits. (E) Protein abundance of GLUT1, PDK1, and LDHA was assessed by western blotting. Student's *t*‐test was adopted for statistical analysis. ***p < .001.
**Figure S3 SH2B1 promoted growth and cell cycle progression of NSCLC cells via interaction with IRS1**. (A) The interaction between SH2B1 and IRS1/JAK2 proteins in NSCLC cells was validated by Co‐IP assay. JAK2 is a known binding partner of SH2B1. (B) The direct interaction between SH2B1 and IRS1 proteins was evaluated by a GST pull‐down assay. NSCLC cells were transfected with sh‐IRS1 together with or without SH2B1 overexpression plasmid for 48 h. (C) Western blotting analysis of SH2B1 and IRS1 protein levels in NSCLC cells. (D, E) The growth of NSCLC cells was analyzed by EdU staining (D) and CCK‐8 assay (E). Scale bar = 100 µm in (D). (F) PI staining combined with flow cytometry evaluated NSCLC cell cycle progression. ANOVA followed by Tukey's test was adopted for statistical analysis. *p < .05, **p < .01, and ***p < .001.
**Figure S4 SH2B1 promoted glycolysis of NSCLC cells via interaction with IRS1**. NSCLC cells were transfected with sh‐IRS1 together with or without SH2B1 overexpression plasmid for 48 h. (A–D) Glucose uptake (A), lactate production (B), ECAR (C), and OCR (D) of NSCLC cells were assessed by commercial kits, respectively. (E) The protein levels of GLUT1, PDK1, LDHA, p‐PI3K, PI3K, p‐AKT and AKT were determined by western blotting. ANOVA followed by Tukey's test was adopted for statistical analysis. *p < .05, **p < .01, and ***p < .001.
**Figure S5 KLF13 repressed the glycolysis of NSCLC cells via transcriptional inhibition of SH2B1**. NSCLC cells were transfected with the KLF13 overexpression plasmid, combined with or without the SH2B1 overexpression plasmid. (A) Western blotting analyzed the protein levels of KLF13, SH2B1, IRS1, p‐PI3K, PI3K, p‐AKT, and AKT in NSCLC cells. (B) PI staining combined with flow cytometry determined NSCLC cell cycle progression. (C) The proliferation of NSCLC cells was detected by EdU staining. Scale bar = 100 µm. (D–G) Glucose uptake (D), lactate production (E), ECAR (F), and OCR (G) of NSCLC cells were measured by commercial kits, respectively. (H) GLUT1, PDK1, and LDHA protein levels were analyzed by western blotting. ANOVA followed by Tukey's test was adopted for statistical analysis. *p < .05, **p < .01, and ***p < .001.
**Figure S6 Exosomal miR‐3126‐5p targeted KLF13 to accelerate growth and cell cycle progression of NSCLC cells**. NSCLC cells were treated with Exos (1 × 10^11^ particles/mL) isolated from CAFs from different groups, combined with or without sh‐KLF13 transfection. (A) MiR‐3126‐5p expression in NSCLC cells was detected by RT‐qPCR. (B) KLF13, SH2B1, and IRS1 protein levels were measured by western blotting. (C, D) NSCLC cell cycle progression was evaluated by PI staining combined with flow cytometry. (E, F) The growth of NSCLC cells was determined by EdU staining. Scale bar = 100 µm. ANOVA followed by Tukey's test was adopted for statistical analysis. *p < .05, **p < .01, and ***p < .001.
**Figure S7 CAFs‐derived Exos promoted glycolysis to drive NSCLC growth in vivo by decreasing KLF13 expression**. The C57BL/6N mice were subcutaneously injected with LLC cells infected with lentiviruses carrying vector or KLF13 in combination with tail vein injection of CAFs‐derived Exos (5 × 10^10^ particles/mouse in 100 µL PBS). (A) The image for tumours. (B) Tumour volume and (C) tumour weight were monitored. (D) Glucose uptake and (E) lactate production in tumour tissues were detected by commercial kits. (F) MiR‐3126‐5p expression in tumours was evaluated by RT‐qPCR. (G) KLF13, SH2B1, and IRS1 protein levels in tumours were detected by western blotting. ANOVA followed by Tukey's test was adopted for statistical analysis. **p < .01 and ***p < .001.
**Figure S8 CAFs‐derived Exos favoured glycolysis and NSCLC growth in vivo via activation of the PI3K/AKT pathway**. The BALB/c nude mice were subcutaneously injected with A549 or NCI‐H1299 cells in combination with tail vein injection of CAFs‐derived Exos (5 × 10^10^ particles/mouse in 100 µL PBS) together with or without BKM120. (A) The image for xenografts. (B) Tumour volume and (C) tumour weight were monitored. (D) Glucose uptake and (E) lactate production in tumour tissues were measured by commercial kits. (F) MiR‐3126‐5p expression in tumours was analyzed by RT‐qPCR. (G) KLF13, SH2B1, IRS1, p‐PI3K, PI3K, p‐AKT, and AKT protein levels in tumours were assessed by western blotting. ANOVA followed by Tukey's test was adopted for statistical analysis. *p < .05, **p < .01, and ***p < .001.

## Data Availability

The datasets analyzed in this study are available from the corresponding author upon reasonable request.
