# Supplementary material for: Exosomal miR‐3126‐5p derived from cancer‐associated fibroblasts facilitates glycolysis to accelerate NSCLC progression by targeting KLF13 to activate the SH2B1/IRS1 axis
Source: Clin Transl Med. 2025 Dec 18;15(12):e70554. doi: 10.1002/ctm2.70554 (PMC12712867; doi:10.1002/ctm2.70554)
Supplement: Supplementary file 1 — Figure S1 SH2B1 deficiency restrained growth and cell cycle progression of NSCLC cells. (A, B) NSCLC cells were transfected with sh‐SH2B1#1, 2, 3 or SH2B1 overexpression plasmid for 48 h, and RT‐qPCR (A) and western blotting (B) analyses of SH2B1 expression. NSCLC cells were transfected with sh‐SH2B1#2 or SH2B1 overexpression plasmid for 48 h. (C, D) The growth of NSCLC cells was evaluated by EdU staining (C) and CCK‐8 assay (D). Scale bar = 100 µm in C. (E) Cell cycle progression was determined by PI staining and flow cytometry. ANOVA followed by Tukey's test was adopted for statistical analysis. **p < .01 and ***p < .001. Figure S2 SH2B1 knockout repressed the glycolysis of NSCLC cells. (A) Western blotting analysis of SH2B1 protein level in parental and SH2B1‐knockout NSCLC cells. (B) Cell viability was determined by CCK‐8. (C, D) Glucose uptake (C) and lactate production (D) were detected by commercial kits. (E) Protein abundance of GLUT1, PDK1, and LDHA was assessed by western blotting. Student's t‐test was adopted for statistical analysis. ***p < .001. Figure S3 SH2B1 promoted growth and cell cycle progression of NSCLC cells via interaction with IRS1. (A) The interaction between SH2B1 and IRS1/JAK2 proteins in NSCLC cells was validated by Co‐IP assay. JAK2 is a known binding partner of SH2B1. (B) The direct interaction between SH2B1 and IRS1 proteins was evaluated by a GST pull‐down assay. NSCLC cells were transfected with sh‐IRS1 together with or without SH2B1 overexpression plasmid for 48 h. (C) Western blotting analysis of SH2B1 and IRS1 protein levels in NSCLC cells. (D, E) The growth of NSCLC cells was analyzed by EdU staining (D) and CCK‐8 assay (E). Scale bar = 100 µm in (D). (F) PI staining combined with flow cytometry evaluated NSCLC cell cycle progression. ANOVA followed by Tukey's test was adopted for statistical analysis. *p < .05, **p < .01, and ***p < .001. Figure S4 SH2B1 promoted glycolysis of NSCLC cells via interaction with IRS1. NSCLC cells [file CTM2-15-e70554-s001.docx]

**Supplementary figures and legends:**

**
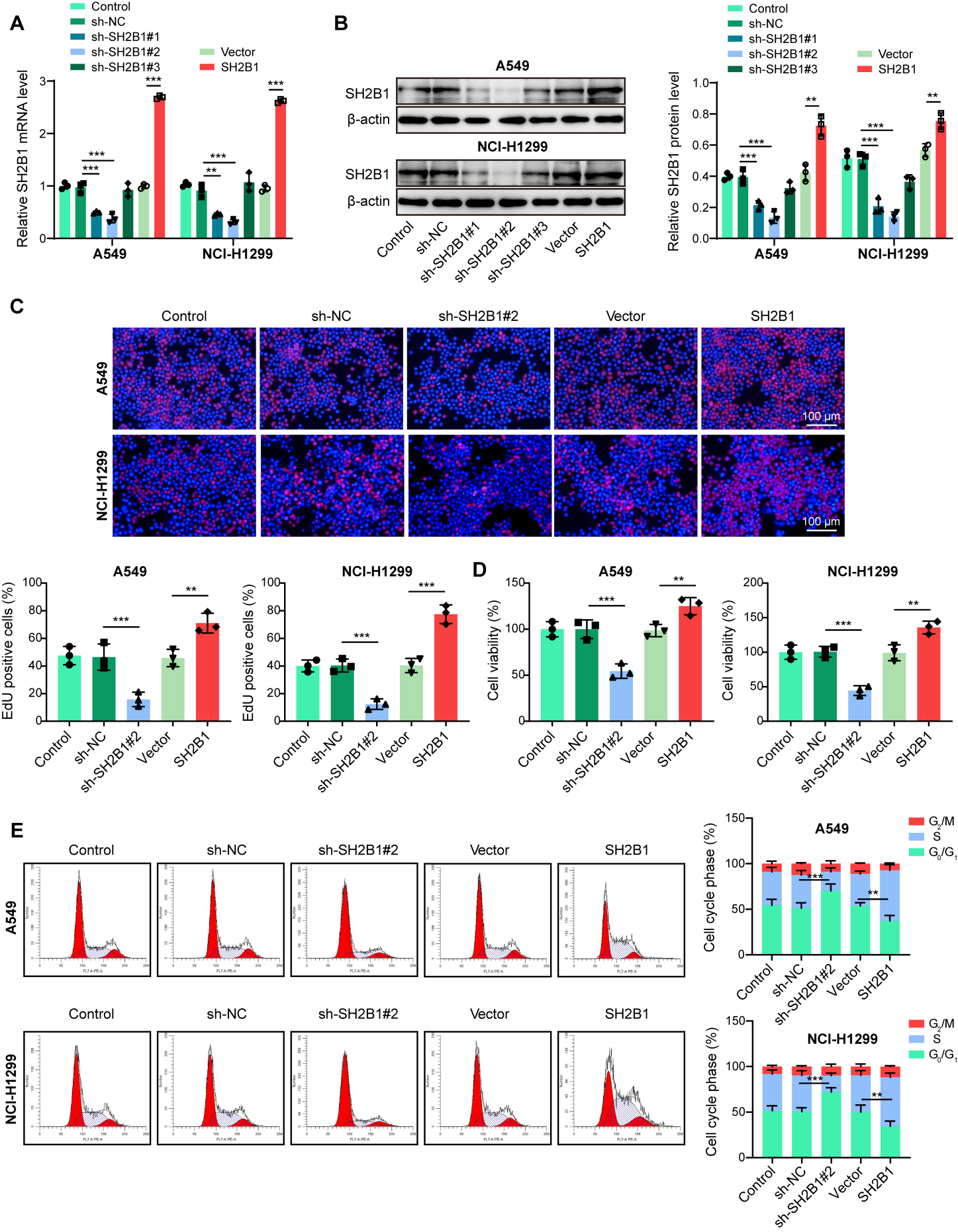
**

**Figure S1 SH2B1 deficiency restrained growth and cell cycle progression of NSCLC cells.**

(A-B) NSCLC cells were transfected with sh-SH2B1#1, 2, 3 or SH2B1 overexpression plasmid for 48 h, and RT-qPCR (A) and western blotting (B) analyses of SH2B1 expression. NSCLC cells were transfected with sh-SH2B1#2 or SH2B1 overexpression plasmid for 48 h. (C-D) The growth of NSCLC cells was evaluated by EdU staining (C) and CCK-8 assay (D). Scale bar=100 μm in C. (E) Cell cycle progression was determined by PI staining and flow cytometry. ANOVA followed by Tukey’s test was adopted for statistical analysis. ***P*<0.01 and ****P*<0.001.

**
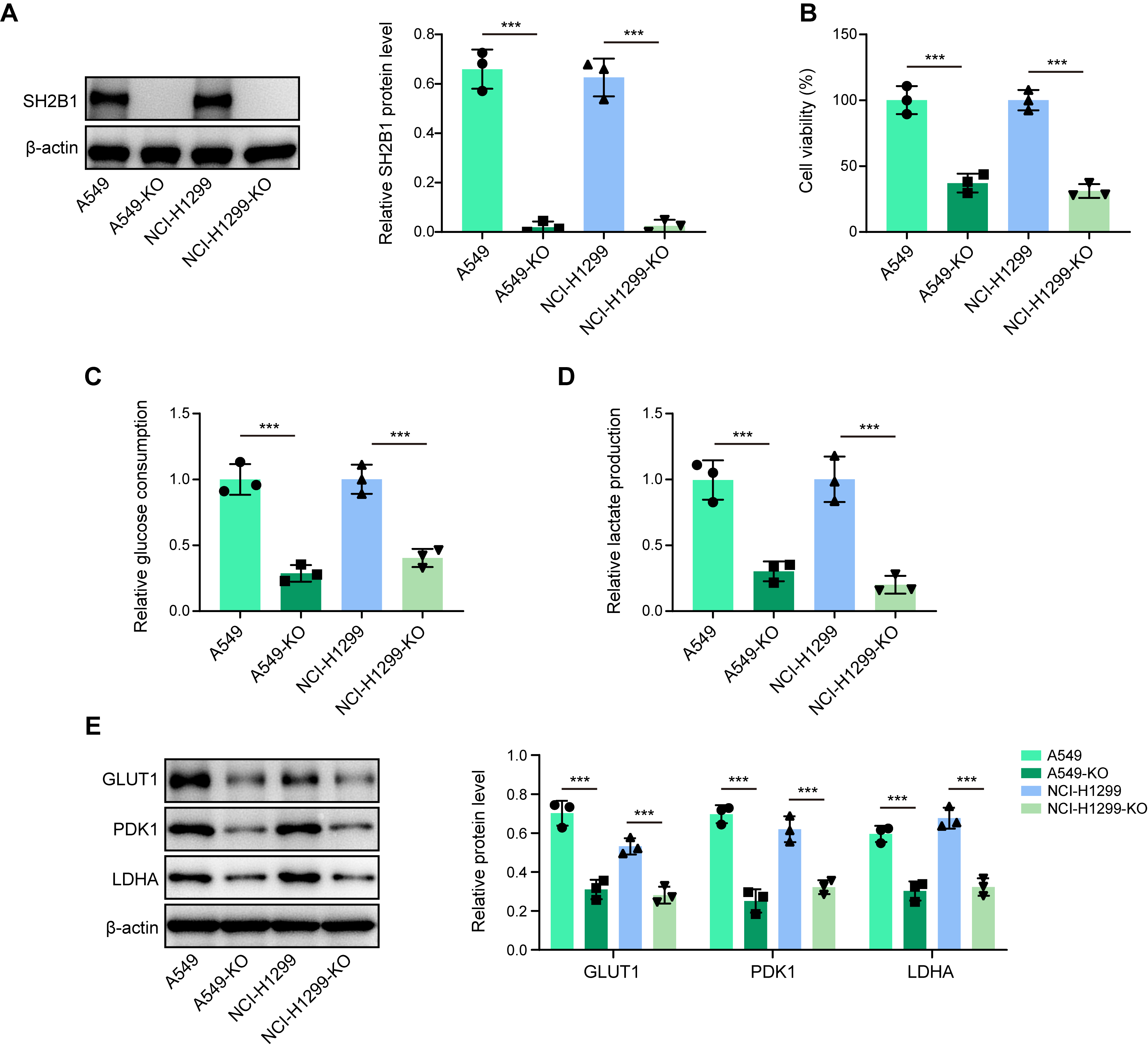
**

**Figure S2 SH2B1 knockout repressed glycolysis of NSCLC cells.**

(A) Western blotting analysis of SH2B1 protein level in parental and SH2B1-konckout NSCLC cells. (B) Cell viability was determined by CCK-8. (C-D) Glucose uptake (C) and lactate production (D) were detected by commercial kits. (E) Protein abundance of GLUT1, PDK1, and LDHA was assessed by western blotting. Student’s t test was adopted for statistical analysis. ****P*<0.001.


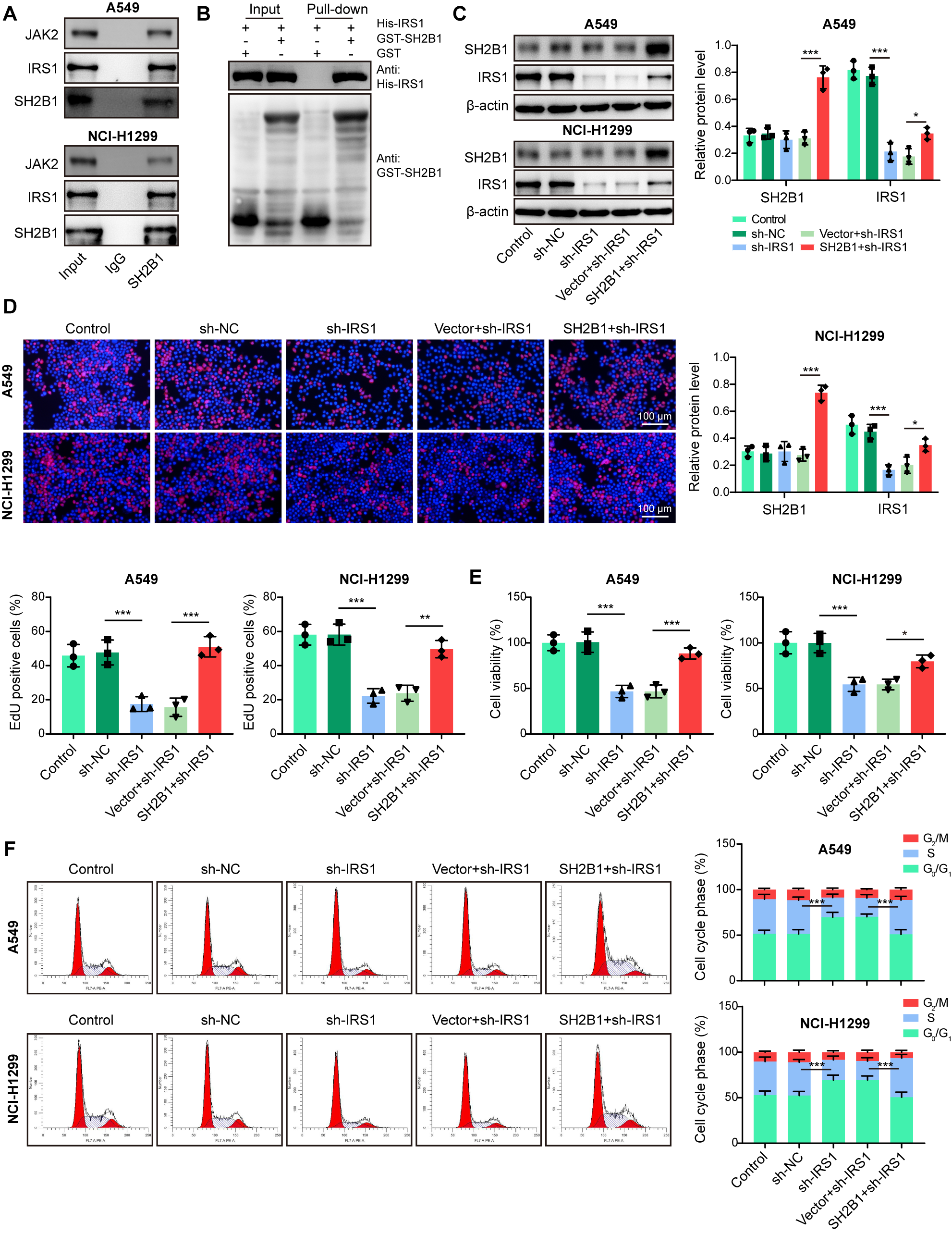


**Figure S3 SH2B1 promoted growth and cell cycle progression of NSCLC cells via interaction with IRS1.**

(A) The interaction between SH2B1 and IRS1/JAK2 proteins in NSCLC cells was validated by Co-IP assay. JAK2 as a known binding partner of SH2B1. (B) The direct interaction between SH2B1 and IRS1 proteins was evaluated by GST pull-down assay. NSCLC cells were transfected with sh-IRS1 together with or without SH2B1 overexpression plasmid for 48 h. (C) Western blotting analysis of SH2B1 and IRS1 protein levels in NSCLC cells. (D-E) The growth of NSCLC cells was analyzed by EdU staining (D) and CCK-8 assay (E). Scale bar=100 μm in D. (F) PI staining combined with flow cytometry evaluated NSCLC cell cycle progression. ANOVA followed by Tukey’s test was adopted for statistical analysis. **P*<0.05, ***P*<0.01, and ****P*<0.001.

**
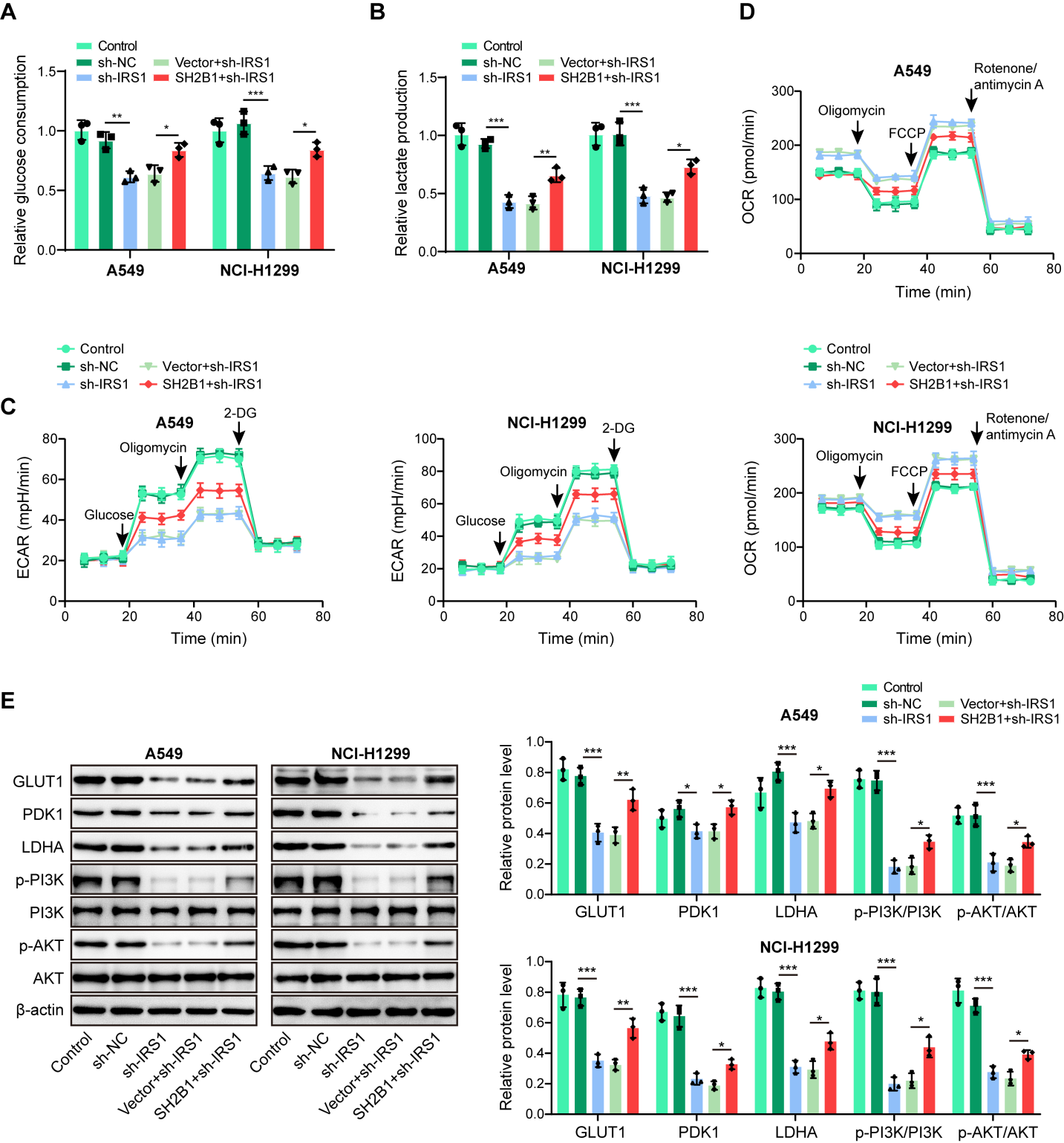
**

**Figure S4 SH2B1 promoted glycolysis of NSCLC cells via interaction with IRS1.**

NSCLC cells were transfected with sh-IRS1 together with or without SH2B1 overexpression plasmid for 48 h. (A-D) Glucose uptake (A), lactate production (B), ECAR (C), and OCR (D) of NSCLC cells were assessed by commercial kits, respectively. (E) The protein levels of GLUT1, PDK1, LDHA, p-PI3K, PI3K, p-AKT and AKT were determined by western blotting. ANOVA followed by Tukey’s test was adopted for statistical analysis. **P*<0.05, ***P*<0.01, and ****P*<0.001.

**
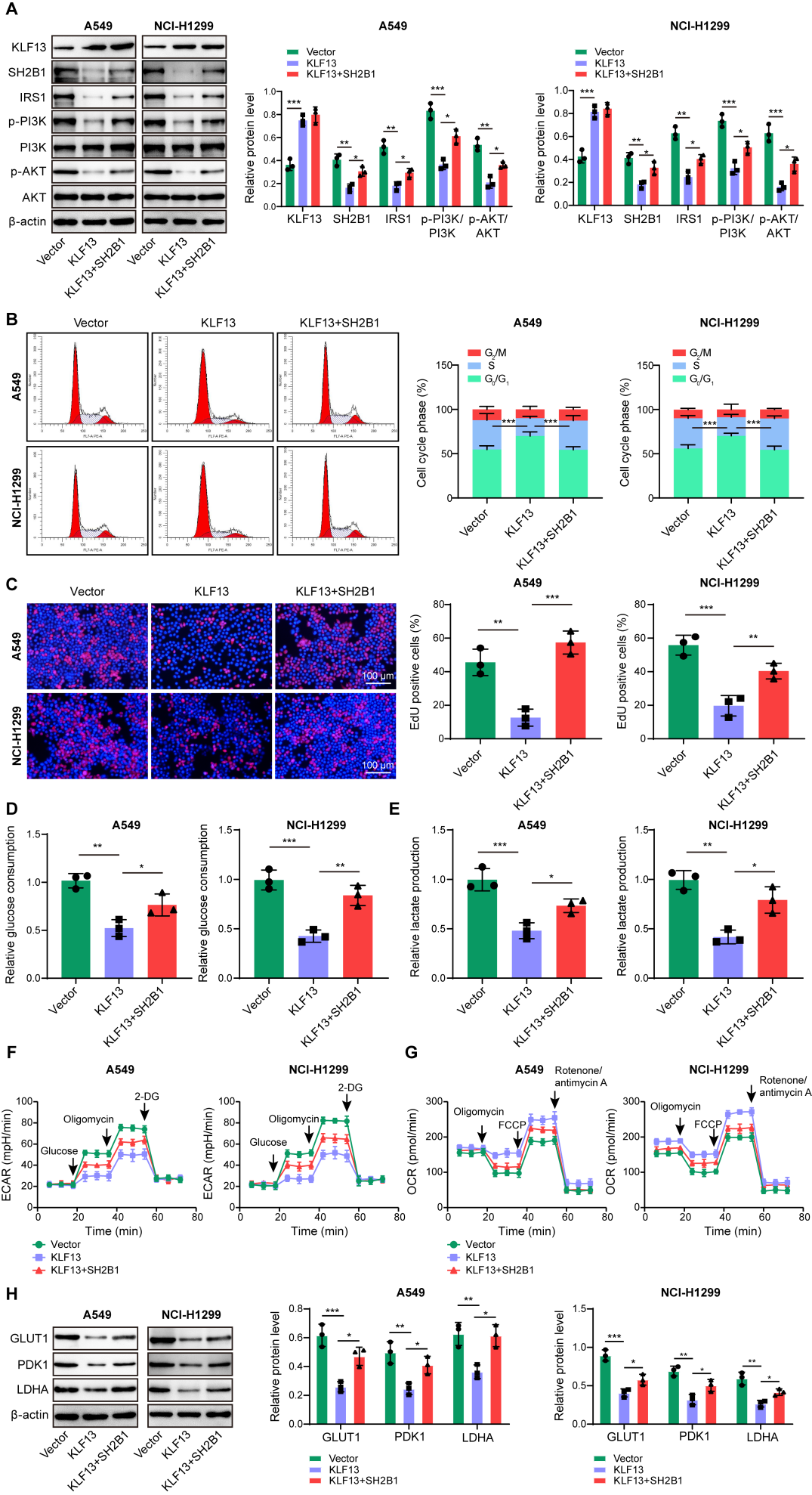
**

**Figure S5 KLF13 repressed glycolysis of NSCLC cells via transcriptional inhibition of SH2B1.** NSCLC cells were transfected with KLF13 overexpression plasmid combined with or without SH2B1 overexpression plasmid. (A) Western blotting analyzed the protein levels of KLF13, SH2B1, IRS1, p-PI3K, PI3K, p-AKT, and AKT in NSCLC cells. (B) PI staining combined with flow cytometry determined NSCLC cell cycle progression. (C) The proliferation of NSCLC cells was detected by EdU staining. Scale bar=100 μm. (D-G) Glucose uptake (D), lactate production (E), ECAR (F), and OCR (G) of NSCLC cells were measured by commercial kits, respectively. (H) GLUT1, PDK1, and LDHA protein levels were analyzed by western blotting. ANOVA followed by Tukey’s test was adopted for statistical analysis. **P*<0.05, ***P*<0.01, and ****P*<0.001.


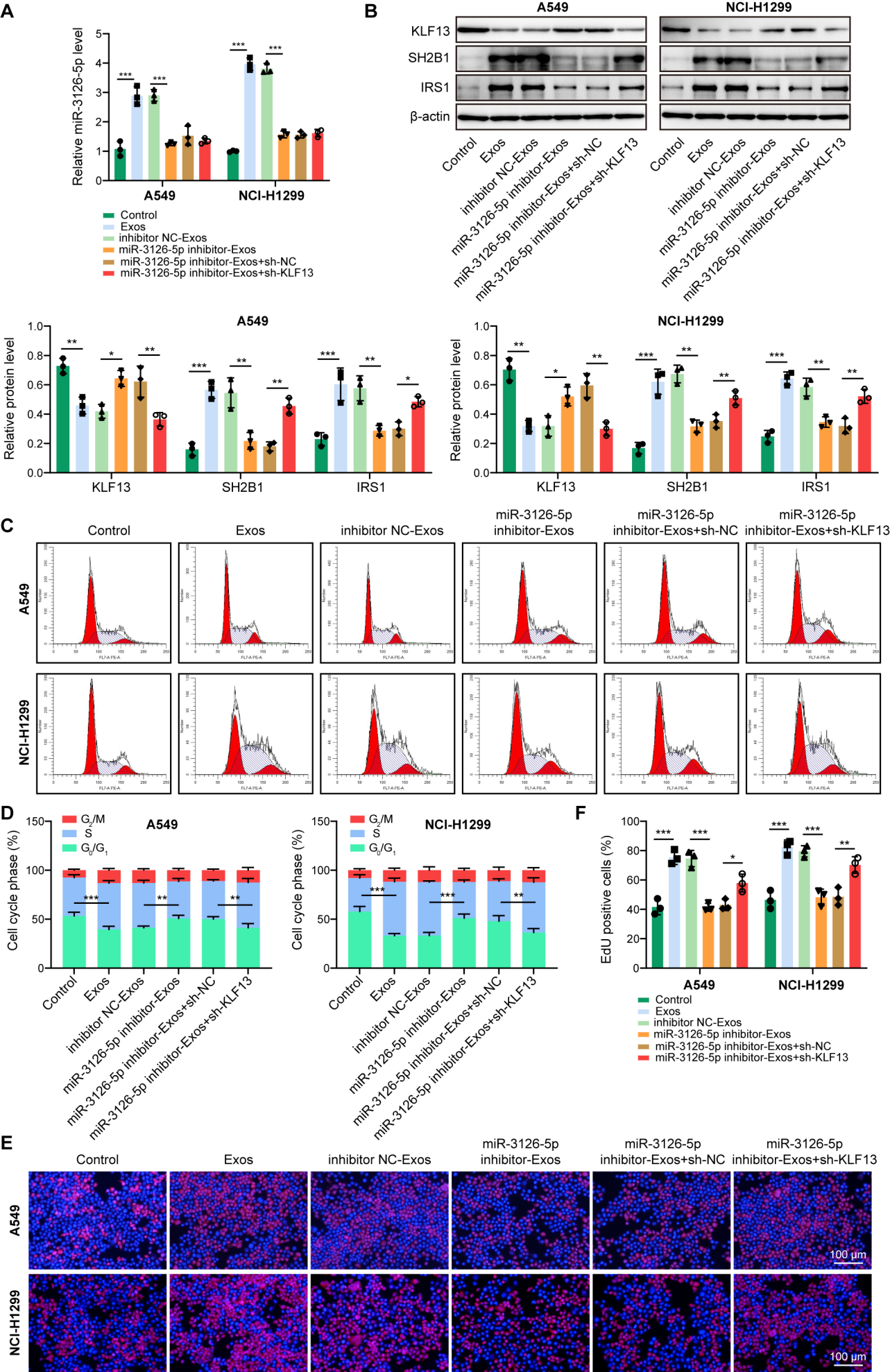


**Figure S6 Exosomal miR-3126-5p targeted KLF13 to accelerate growth and cell cycle progression of NSCLC cells.**

NSCLC cells were treated with Exos (1×10^11^ particles/mL) isolated from CAFs from different groups combined with or without sh-KLF13 transfection. (A) MiR-3126-5p expression in NSCLC cells was detected by RT-qPCR. (B) KLF13, SH2B1, and IRS1 protein levels were measured by western blotting. (C-D) NSCLC cell cycle progression was evaluated by PI staining combined with flow cytometry. (E-F) The growth of NSCLC cells was determined by EdU staining. Scale bar=100 μm. ANOVA followed by Tukey’s test was adopted for statistical analysis. **P*<0.05, ***P*<0.01, and ****P*<0.001.


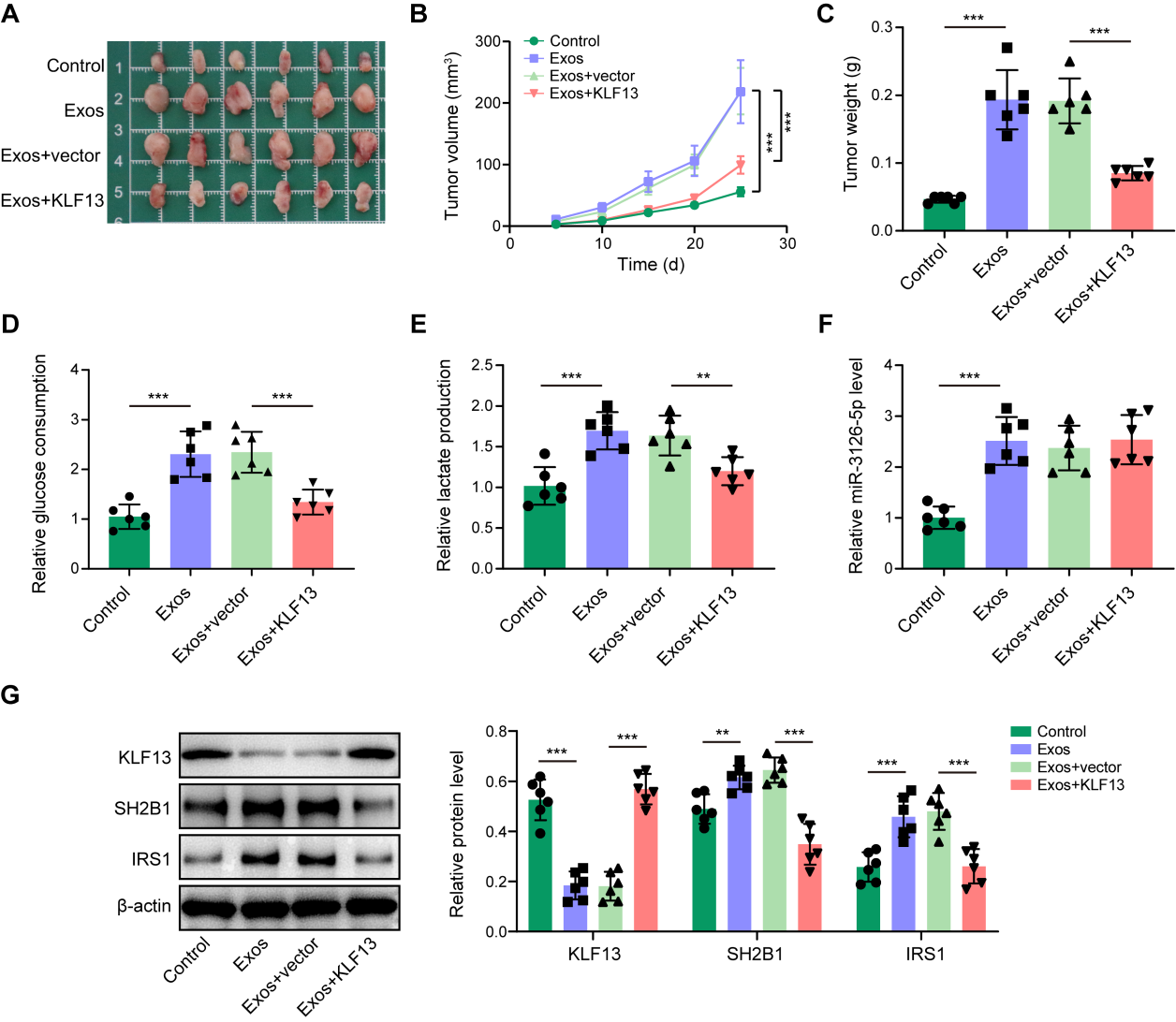


**Figure S7 CAFs-derived Exos promoted glycolysis to drive NSCLC growth *in vivo* by decreasing KLF13 expression.**

The C57BL/6N mice were subcutaneously injected with LLC cells infected with lentiviruses carrying vector or KLF13 in combination with tail vein injection of CAFs-derived Exos (5×10^10^ particles/mouse in 100 μL PBS). (A) The image for tumors. (B) Tumor volume and (C) tumor weight were monitored. (D) Glucose uptake and (E) lactate production in tumor tissues were detected by commercial kits. (F) MiR-3126-5p expression in tumors was evaluated by RT-qPCR. (G) KLF13, SH2B1, and IRS1 protein levels in tumors were detected by western blotting. ANOVA followed by Tukey’s test was adopted for statistical analysis. ***P*<0.01 and ****P*<0.001.

**
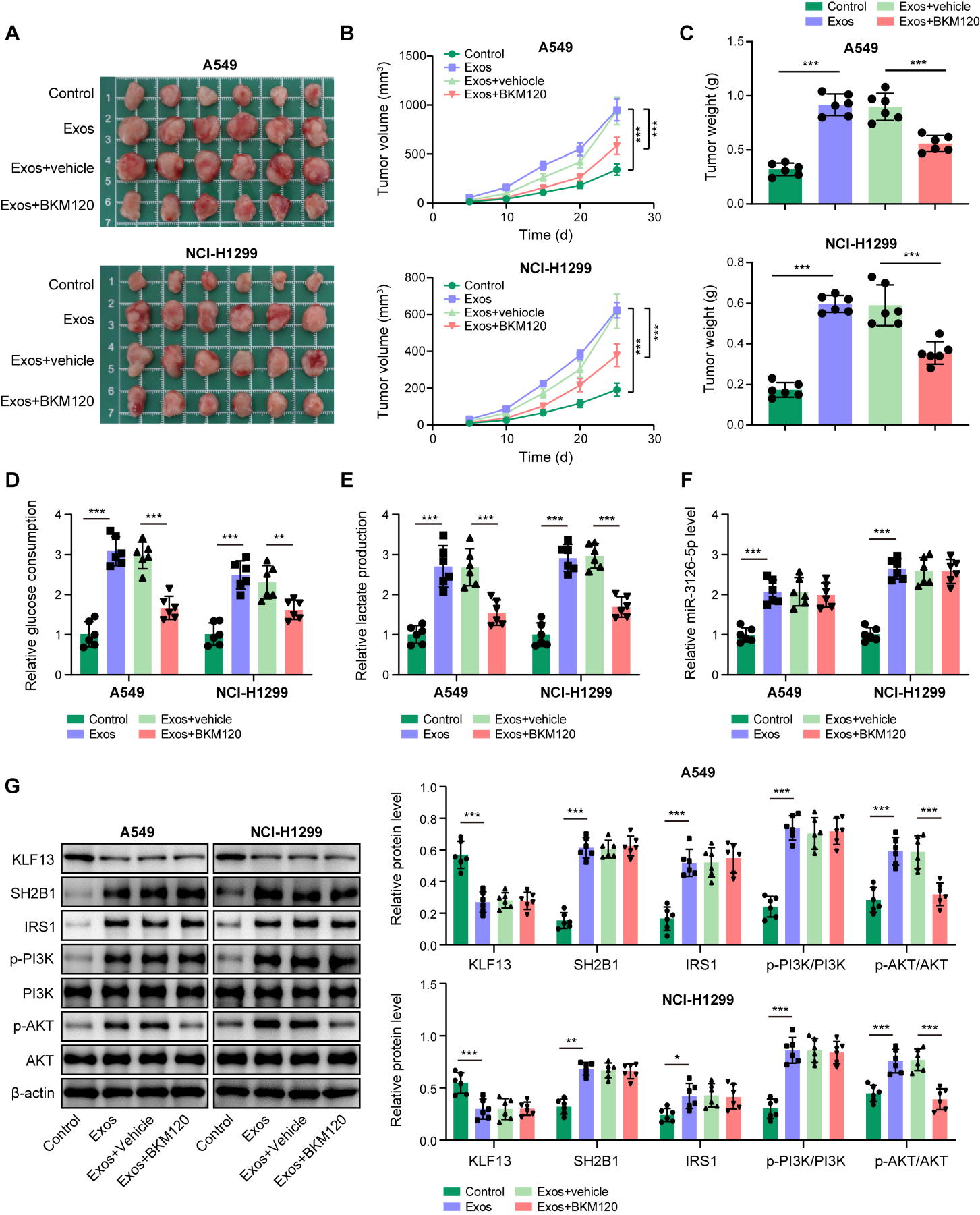
**

**Figure S8 CAFs-derived Exos favored glycolysis and NSCLC growth *in vivo* via activation of PI3K/AKT pathway.**

The BALB/c nude mice were subcutaneously injected with A549 or NCI-H1299 cells in combination with tail vein injection of CAFs-derived Exos (5×10^10^ particles/mouse in 100 μL PBS) together with or without BKM120. (A) The image for xenografts. (B) Tumor volume and (C) tumor weight were monitored. (D) Glucose uptake and (E) lactate production in tumor tissues were measured by commercial kits. (F) MiR-3126-5p expression in tumors was analyzed by RT-qPCR. (G) KLF13, SH2B1, IRS1, p-PI3K, PI3K, p-AKT, and AKT protein levels in tumors were assessed by western blotting. ANOVA followed by Tukey’s test was adopted for statistical analysis. **P*<0.05, ***P*<0.01, and ****P*<0.001.
